# Neural oscillations during cognitive processes in an *App* knock-in mouse model of Alzheimer’s disease pathology

**DOI:** 10.1038/s41598-019-51928-w

**Published:** 2019-11-08

**Authors:** Sofia Jacob, Gethin Davies, Marijke De Bock, Bart Hermans, Cindy Wintmolders, Astrid Bottelbergs, Marianne Borgers, Clara Theunis, Bianca Van Broeck, Nikolay V. Manyakov, Detlef Balschun, Wilhelmus H.I.M. Drinkenburg

**Affiliations:** 1 Department of Neuroscience, Janssen Research & Development, a Division of Janssen Pharmaceutica NV, Beerse, Belgium; 2 Brain & Cognition, KU Leuven, Leuven, Belgium; 3 Digital Phenotyping, Janssen Research & Development, a Division of Janssen Pharmaceutica NV, Beerse, Belgium; 4 Groningen Institute for Evolutionary Life Sciences, University of Groningen, Groningen, The Netherlands

**Keywords:** Alzheimer's disease, Dynamic networks

## Abstract

Multiple animal models have been created to gain insight into Alzheimer’s disease (AD) pathology. Among the most commonly used models are transgenic mice overexpressing human amyloid precursor protein (APP) with mutations linked to familial AD, resulting in the formation of amyloid β plaques, one of the pathological hallmarks observed in AD patients. However, recent evidence suggests that the overexpression of APP by itself can confound some of the reported observations. Therefore, we investigated in the present study the *App*^*NL-G-F*^model, an *App* knock-in (*App*-KI) mouse model that develops amyloidosis in the absence of APP-overexpression. Our findings at the behavioral, electrophysiological, and histopathological level confirmed an age-dependent increase in Aβ1–42 levels and plaque deposition in these mice in accordance with previous reports. This had apparently no consequences on cognitive performance in a visual discrimination (VD) task, which was largely unaffected in *App*^*NL-G-F*^ mice at the ages tested. Additionally, we investigated neurophysiological functioning of several brain areas by phase-amplitude coupling (PAC) analysis, a measure associated with adequate cognitive functioning, during the VD task (starting at 4.5 months) and the exploration of home environment (at 5 and 8 months of age). While we did not detect age-dependent changes in PAC during home environment exploration for both the wild-type and the *App*^*NL-G-F*^ mice, we did observe subtle changes in PAC in the wild-type mice that were not present in the *App*^*NL-G-F*^ mice.

## Introduction

Alzheimer’s disease (AD) is the most common form of progressive neurodegenerative dementia^1^. Considering that the main risk factor of the disease is age^2^ and that average life-expectancy increases, the associated personal, social and socio-economic burden will intensify if we do not find an effective treatment. Two major pathological hallmarks of AD are amyloid beta (Aβ) senile plaques and tau neurofibrillary tangles (NFTs)^1^. These changes are accompanied by neuroinflammation, aberrant synaptic and neuronal network activities^3–5^, and eventually dramatic brain shrinkage due to neuronal damage^1^. A clear understanding of the specific role of Aβ plaques and NFTs and their interaction in (functional) aspects of the disease remains a topic of investigation. Available evidence suggests a model of AD progression with three phases: preclinical AD, mild cognitive impairment (MCI), and clinical AD. The preclinical AD starts around 20 years before AD full symptoms’ onset and is characterized by Aβ accumulation in the cortex without any neurological symptoms. During MCI some cognitive symptoms as well as tauopathy start to emerge. At the clinical AD phase, severe symptoms of dementia are accompanied by irreversible neurodegeneration^6,7^. It is presently believed that the best chance of therapeutic success in AD may be early intervention during the preclinical AD phase.

Numerous animal models mirroring some features of AD pathogenesis have been created to facilitate the understanding of the molecular mechanisms of the disease^8^. Among the most common models are transgenic mouse models overexpressing the human amyloid precursor protein (APP) with mutations linked to familial AD in APP or/and presenilin 1 and 2^7^. Although these animals have been instrumental in understanding some basic aspects of AD^7^, the overexpression of these proteins might lead to artificial phenotypes^9,10^. At the brain network level, it has been reported by numerous studies that these mouse models demonstrate various alterations^11–13^, even before Aβ accumulation^14^. Although these results show some phenotypical similarities with AD^3,15,16^, it is not clear what are the underlying mechanisms that cause the aberrant neuronal activity observed in these models. Some evidence suggests that APP overexpression, and not Aβ overproduction might be responsible for the abnormal network activity in APP-overexpressing mouse models^10^. Further evidence supporting this hypothesis, comes from studies investigating the physiological role of APP or its processed fragments in non-pathological conditions^17,18^. For instance, it has been demonstrated that sAPPα (one of the APP fragments) plays a role in the modulation of synaptic transmission^17^.

In 2014, Saito and colleagues developed a new generation of APP Knock-In (KI) mice that produce robust Aβ amyloidosis with physiological APP levels^9,19^. These mice express humanized Aβ with either one (Swedish, *App*^*NL*^), two (Swedish and Beyreuther/Iberian, *App*^*NL-F*^), or three (Swedish, Beyreuther/Iberian, and Artic, *App*^*NL-G-F*^) familial AD mutations. While Aβ plaque deposition starts at 6 months in *App*^*NL-F*^ mice^19^, its onset in *App*^*NL-G-F*^ mice is already at 2 months and saturates around 7 months^19,20^. In contrast, *App*^*NL*^ mice do not develop any Aβ plaques, even at late ages^19,20^. Behaviorally, *App*^*NL-G-F*^ mice demonstrate impairments in multiple cognitive domains, including declined spatial reversal learning and attention control, loss of avoidance memory, and enhanced impulsivity and compulsivity, starting at 8 months of age^21^. Studies investigating early time points show more inconclusive results. For instance, while in the original report, deficits in the Y-maze were indicated^19^, a later study did not find deficits in working memory using the Y-maze at 6 months of age^22^. Another study testing *App*^*NL-G-F*^ mice at 3, 6, and 10 months demonstrated largely unaffected behavioral readouts, with only mild changes in social and anxiety-related test performance^23^. Brown *et al*., investigated network hyperexcitability in two mouse models: *App*^*NL-F*^and J20 (APP-overexpression). Although they were able to replicate the previously reported effect on network hyperexcitability in the J20 mice, the effect was absent in *App*^*NL-F*^ mice^24^.

The *App*-KI models have not yet been fully characterized with respect to neuronal network activity. Therefore, the primary goal of this study was to investigate electrophysiological readouts in combination with a cognitive task to illuminate functional differences between *App*^*NL-G-F*^ mice and wild-type C57BL/6J (WT) controls. Mice had to perform a visual discrimination (VD) task using touch-screen operant boxes, starting at 4.5 months of age while local field potentials (LFPs) in the dorsal medial striatum (DMS), cingulate cortex (Cg), retrosplenial cortex (RSC), and dorsal CA1 (dCA1) region of the hippocampus were recorded using a wireless neurophysiological signal acquisition system. This approach has multiple advantages: Firstly, the use of a wireless LFP recording system allowed the mice to move freely in the operant box. Secondly, the touch-screen platform permitted a high level of standardization and lowered the motoric demands on the mice^25^. Finally, the synchronization of LFPs with different behavioral parameters enabled a precise quantification of electrophysiological changes related to behavioral performance; thus, making optimal use of the high temporal resolution of LFPs recording techniques. In parallel with these recordings, we measured LFPs at two time-points during home-cage exploration, without the behavioral task to investigate neuronal network changes exclusively associated with pathology progression. The ages to perform the *in vivo* experiments were selected with the objective to match the preclinical AD phase. Biochemical and immunohistochemical analysis were conducted to correlate Aβ plaque deposition with the electrophysiological and behavioral results at the different time-points. The electrophysiological analysis focussed on phase-amplitude coupling (PAC) between theta (4–12 Hz), gamma (30–100 Hz), and high-frequency oscillations (100–200 Hz, HFO) activity during three distinct recording conditions related to different cognitive load, the start and the end of the VD task, and exploration of the home environment. PAC has been suggested to be a potential mechanism to regulate neuronal communication in multiple brain regions^26–28^. Furthermore, neuronal oscillations are affected in pathological conditions. For instance, neurodegenerative diseases are characterized by, among others, a disruption of gamma oscillations, which in turn is associated with the cognitive deficits^3^. In addition to new insights into pathological processes of this second-generation mouse model of Aβ amyloid pathology, our approach provides a versatile tool to further assess the complex interplay between different frequency bands and their relationship with behavior. The combination of these techniques may therefore open new avenues for the investigation of cognition-related network perturbations in relevant animal models of AD pathology, eventually determining their translational validity and potential use as biomarkers in drug discovery and development.

## Results

### Performance during the visual discrimination (VD) task

#### Pre-training

During the pre-training stage there were no genotype effects on the number of sessions required in the tone association (WT: *M* = 3.9, *SD* = 0.7, *n* = 10; *App*^*NL-G-F*^: *M* = 4.5, *SD* = 0.5, *n* = 8, Z = 3.74, *p* = 0.05), touch association (WT: *M* = 1.2, SD = 0.6, *n* = 10; *App*^*NL-G-F*^: *M* = 1.0, *SD* = 0.0, *n* = 8, Z = 0.8, *p* = 0.37), must touch (WT_:_ *M* = 1.4, *SD* = 0.8, *n* = 10; *App*^*NL-G-F*^: *M* = 1.4, *SD* = 1.1, *n* = 8, Z = 0.07, *p* = 0.78) and punish incorrect (WT: *M* = 3.9, *SD* = 1.4, *n* = 10; *App*^*NL-G-F*^: *M* = 4.3, *SD* = 1.4, *n* = 7, Z = 0.31, *p* = 0.57) stages of touch-screen pre-training. One *App*^*NL-G-F*^ mouse did not complete all pre-training stages within 30 days and did not progress onto the VD task.

#### Discrimination task

**Figure 1:**
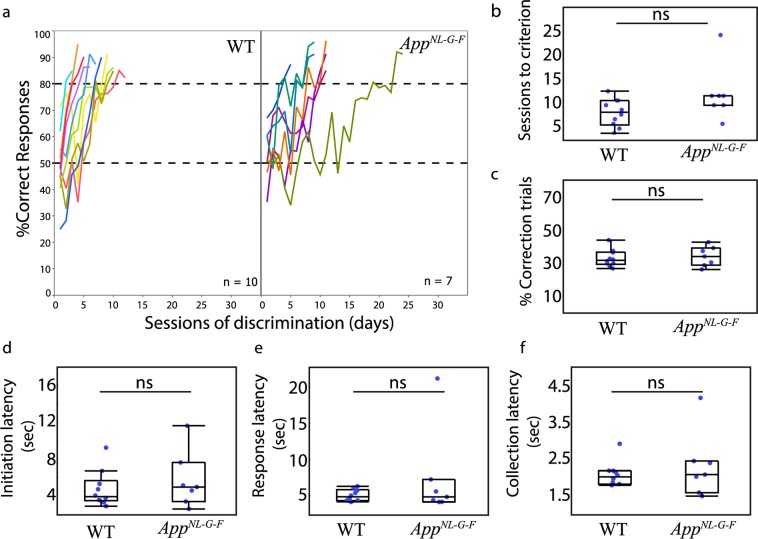
Acquisition of the visual discrimination (VD) touch-screen task in *App*^*NL-G-F*^ and WT mice. (**a**) Learning curves of mice during discrimination learning for WT (left panel) and *App*^*NL-G-F*^ (right panel) plotting percentage correct responses by sessions where each colored line represents an individual mouse. (**b)** Number of sessions to achieve learning criterions of two consecutive sessions of 80% correct or higher responses did not differ between WT and *App*^*NL-G-F*^ mice. (**c)** Percentage of correction trials did not differ between genotypes. Latencies, (**d)** to initiate trial, (**e)** response to the stimulus, (**f)** collect the reward did not differ between genotypes. In figures b, c, d, e, f data are represented in box plots with individual points for each subject. Statistically significance level at p < 0.05. ns: non-significant.

**Figure 2:**
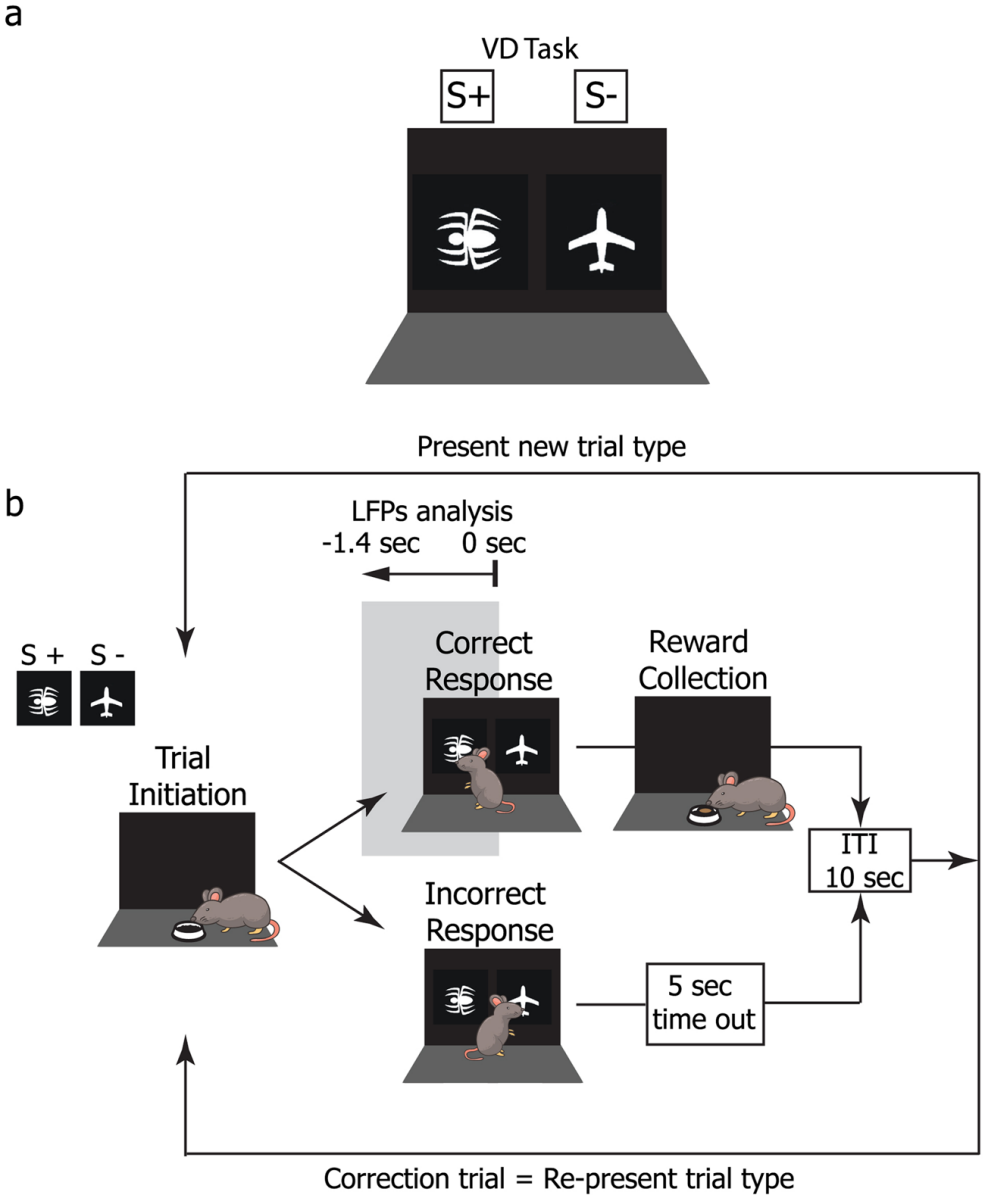
Visual discrimination (VD) touch-screen task. (**a)** The spider/plane stimulus image pair used in the VD^25^. In this example, the spider was the rewarded image (*conditioned stimulus:* S+) and the plane the unrewarded (*unconditioned stimulus:* S−) image. Image contingency was counterbalanced across animals. (**b)** Illustration of the VD task. A trial began with the delivery of a food pellet in the food magazine opposite to the touchscreen. Collection of the pellet initiated the trial where the two images appeared on the touchscreen. Nose-poking on the S+ stimulus activated the delivery of a food reward and the trial was registered as a correct response. Nose-poking the S− stimulus did not delivery a food reward, the light of the operant chamber went off for 5 seconds, and the response was registered as incorrect. Incorrect trials were followed by correction trials. Grey area represents local field potentials (LFPs) analysis. Epochs of 1.4 seconds immediately preceding activation of the touch-screen during correct responses were selected for electrophysiological analysis.

The individual learning curves of mice during acquisition of the VD task are shown in Fig. 1a. As expected, percentage of correct responses on the first day of the discrimination task was around chance level (WT: *M* = 49.17%, *SD* = 12.46, *n* = 10; *App*^*NL-G-F*^: *M* = 52.97%, *SD* = 10.61, *n* = 7) and no difference was found between genotypes *t* (15) = 0.656, *p* = 0.52. To compare learning rates, a mixed linear model was used to fit the percentage of correct responses data (R^2^ = 0.715). There was a significant effect of session *F* (1, 139.6) = 252.3, *p* < 0.0001, and a significant interaction of session and genotype *F* (1, 139.6) = 29.07, *p* < 0.0001. The main genotype effect did not reach significance *F* (1, 14.7) = 2.375, *p* = 0.1445. One mouse in the *App*^*NL-G-F*^ group needed 24 sessions to reach the VD criterion, twice that of the slowest learner in the WT group. Excluding this extreme value from the model fit resulted in a non-significant interaction term between genotype and session *F* (1, 115.5) = 3.668, *p* = 0.0580. Analysis of the number of sessions required to reach the learning criterion of two consecutive sessions of 80% correct responses or higher did not identify a significant difference between groups (WT: *M* = 7.4%, *SD* = 2.91, *n* = 10; *App*^*NL-G-F*^: *M* = 11.43%, *SD* = 5.94, *n* = 7, Z = 2.95, *p* = 0.08, Fig. 1b). Similarly, a comparison of the percentage of correction trials (CTs) did not indicate a genotype difference (WT: *M* = 32.24%, *SD* = 5.24, *n* = 10; *App*^*NL-G-F*^: *M* = 33.54%, *SD* = 6.08, *n* = 7, Z = 0.15, *p* = 0.69, Fig. 1c). Analysis of latencies to initiate trials (WT: *M* = 4.54%, *SD* = 1.86, *n* = 10; *App*^*NL-G-F*^: *M* = 5.51%, *SD* = 2.9, *n* = 7, Z = 0.61, *p* = 0.43, Fig. 2d), response to the stimulus (WT: *M* = 5.07%, *SD* = 0.87, *n* = 10; *App*^*NL-G-F*^: *M* = 7.69%, *SD* = 6.07, *n* = 7, Z = 0.95, *p* = 0.33, Fig. 1e), and to collect the food reward (WT: *M* = 2.04%, *SD* = 0.34, *n* = 10; *App*^*NL-G-F*^: *M* = 2.28%, *SD* = 0.89, *n* = 7, Z = 0.15, *p* = 0.69, Fig. 1f) indicated no alterations in any response latencies for the *App*^*NL-G-F*^ mice. Together these results suggest that the ability to discriminate visual stimuli and to form stimulus-reward associations was largely unaffected in the *App*^*NL-G-F*^ mice.

### Analysis of brain oscillations during the VD task

#### Relative Power Spectral Density (PSD)

**Figure 3:**
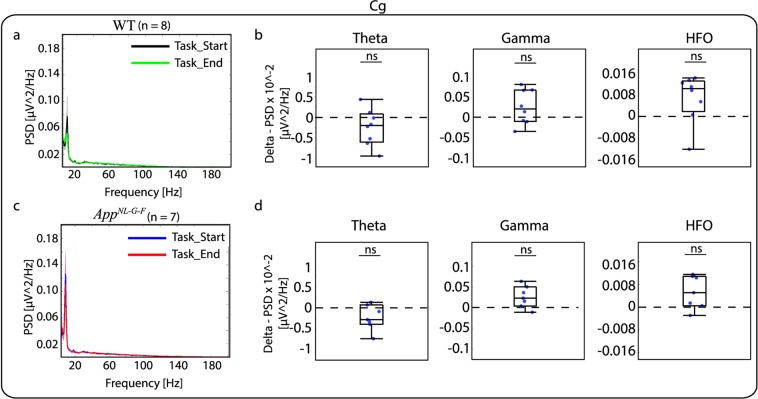
Mean relative power spectral density (PSD) during the visual discrimination (VD) task for the cingulate cortex (Cg). Mean relative PSD in the 4 to 200 Hz band during start of the VD task (Task_Start, black and blue curves) and end of the VD task (Task_End, green and red curves) for (**a**) WT and (**c**) *App*^*NL-G-F*^ mice. Delta between Task_End and Task_Start is represented in box plots were individual points indicate delta values for each subject for (**b**) WT and (**d**) *App*^*NL-G-F*^ mice. No significant difference was observed between the end and the start of the task for the two genotypes at the three different frequency bands. Significance level at *q* = 0.05, false discovery rate (FDR) corrected for multiple comparisons. ns: non-significant.

Before investigating the relative PSD in the four brain areas of interest during the start and end of the VD task, we grouped frequencies on three bands: theta (4 to 12 Hz), gamma (30 to 100 Hz) and HFO (101 to 200 Hz), based on previous proposed guidelines^29^. A within-subject analysis between the end and start of the VD task revealed no significant difference in relative PSD for any of the frequency bands for both genotypes (see Fig. 3 with Cg brain area as an example and Table 1 for all descriptive statistics and statistical tests). We also investigated relative PSD between WT and *App*^*NL-G-F*^, but no significant differences were found for any of the brain areas and frequency bands (Supplementary Table 1).

**Table 1:** Relative power spectral density (PSD, µV^2^/Hz) during the visual discrimination (VD) task.

Genotype	Brain Area	Freq	Condition	Mean	SD	Median	Range	N	Z	Prob > [Z]	p value rank	FDR threshold
WT	Cg	Theta	Task_Start	0.03879	0.01048	0.04082	0.03418	8	−11	0.1484	13	0.02708
			Task_End	0.03639	0.00094	0.03746	0.02788					
		Gamma	Task_Start	0.00540	0.00110	0.00510	0.00360	8	10	0.1953	16	0.03333
			Task_End	0.00560	0.00100	0.00550	0.00300					
		HFO	Task_Start	0.00120	0.00034	0.00120	0.00078	8	13	0.0781	9	0.01875
			Task_End	0.00130	0.00036	0.00120	0.00082					
*App* ^*NL-G-F*^		Theta	Task_Start	0.04875	0.00666	0.04702	0.01918	7	−10	0.1094	11	0.02292
			Task_End	0.04600	0.00860	0.04278	0.02300					
		Gamma	Task_Start	0.00450	0.00077	0.00460	0.00220	7	12	0.0469	4	0.00833
			Task_End	0.00480	0.00083	0.00530	0.00220					
		HFO	Task_Start	0.00093	0.00020	0.00100	0.00049	7	11	0.0781	8	0.01667
			Task_End	0.00099	0.00022	0.00100	0.00059					
WT	dCA1	Theta	Task_Start	0.04992	0.01000	0.05291	0.03031	10	−1.5	0.9219	23	0.04792
			Task_End	0.04901	0.00896	0.05110	0.02459					
		Gamma	Task_Start	0.00420	0.00062	0.00440	0.00200	10	4.5	0.6953	22	0.04583
			Task_End	0.00440	0.00068	0.00430	0.00190					
		HFO	Task_Start	0.00019	0.00006	0.00020	0.00017	10	25.5	0.0059	1	0.00208
			Task_End	0.00025	0.00013	0.00023	0.00049					
*App* ^*NL-G-F*^		Theta	Task_Start	0.05863	0.00790	0.06198	0.01952	6	−8.5	0.0938	10	0.02083
			Task_End	0.05361	0.00862	0.05439	0.02604					
		Gamma	Task_Start	0.00360	0.00100	0.00330	0.00300	6	9.5	0.0625	5	0.01042
			Task_End	0.00410	0.00120	0.00380	0.00380					
		HFO	Task_Start	0.00026	0.00011	0.00026	0.00031	6	9.5	0.0625	6	0.01250
			Task_End	0.00031	0.00013	0.00032	0.00038					
WT	DMS	Theta	Task_Start	0.06310	0.00684	0.06254	0.02294	8	17	0.0156	2	0.00417
			Task_End	0.06634	0.00559	0.06502	0.01872					
		Gamma	Task_Start	0.00320	0.00076	0.00300	0.00250	8	5	0.5469	20	0.04167
			Task_End	0.00330	0.00064	0.00340	0.00220					
		HFO	Task_Start	0.00042	0.00013	0.00037	0.00043	8	−7	0.3828	19	0.03958
			Task_End	0.00041	0.00012	0.00039	0.00037					
*App* ^*NL-G-F*^		Theta	Task_Start	0.06536	0.00494	0.06540	0.01470	7	−7	0.2969	18	0.03750
			Task_End	0.06330	0.01026	0.06251	0.02893					
		Gamma	Task_Start	0.00320	0.00069	0.00310	0.00210	7	9	0.1563	14	0.02917
			Task_End	0.00360	0.00098	0.00320	0.00250					
		HFO	Task_Start	0.00040	0.00014	0.00039	0.00041	7	7	0.2969	17	0.03542
			Task_End	0.00041	0.00014	0.00036	0.00041					
WT	RSC	Theta	Task_Start	0.04116	0.00835	0.03904	0.02734	9	−13.5	0.1289	12	0.02500
			Task_End	0.03915	0.00799	0.03650	0.02338					
		Gamma	Task_Start	0.00480	0.00088	0.00490	0.00300	9	15.5	0.0742	7	0.01458
			Task_End	0.00510	0.00091	0.00510	0.00280					
		HFO	Task_Start	0.00119	0.00033	0.00125	0.00093	9	19.5	0.0195	3	0.00625
			Task_End	0.00132	0.00031	0.00148	0.00071					
*App* ^*NL-G-F*^		Theta	Task_Start	0.04553	0.01188	0.04353	0.03560	7	−1	0.9375	24	0.05000
			Task_End	0.04530	0.01254	0.04611	0.03356					
		Gamma	Task_Start	0.00460	0.00120	0.00460	0.00370	7	3	0.6875	21	0.04375
			Task_End	0.00470	0.00120	0.00480	0.00330					
		HFO	Task_Start	0.00109	0.00027	0.00098	0.00073	7	9	0.1563	15	0.03125
			Task_End	0.00103	0.00028	0.00110	0.00078					

Brain areas: Cingulate Cortex (Cg), dorsal CA1 region of the hippocampus (dCA1), dorsal medial striatum (DMS), and retrosplenial cortex (RSC).

Frequencies: theta, gamma and high frequency oscillations (HFO).

Conditions: Task_Start = start of the VD task, Task_End = end of the VD task.

Descriptive statistics for PSD (µV^2^/Hz), N: sample size, Z: Wilcoxon rank-sum test.

Significance level at *q* = 0.05, false discovery rate (FSD) corrected for multiple comparisons.

#### Phase-Amplitude Coupling (PAC)

**Figure 4:**
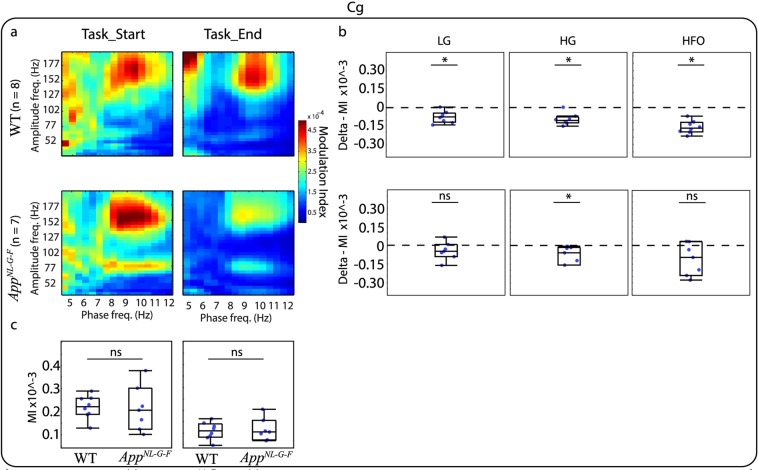
Mean phase-amplitude coupling (PAC) during the visual discrimination (VD) task for the cingulate cortex (Cg). (**a**) Phase-amplitude comodulograms plotted for WT mice (top panels) and *App*^*NL-G-F*^ mice (lower panels) for start of the VD task (Task_Start, left) and end of the VD task (Task_End, right). (**b**) Modulation index (MI) delta between Task_End and Task_Start for low gamma (LG, left), high gamma (HG, middle) and high frequency oscillations (HFO, right) for WT mice (top panels) and *App*^*NL-G-F*^ mice (lower panels). Comparison at each frequency band showed a significant different for WT mice, but only at HG for *App*^*NL-G-F*^ mice. (**c**) MI between WT and *App*^*NL-G-F*^ mice for Task_Start (left) and Task_End (right). Comparison at each frequency band showed no significant difference. Values are represented in box plots were individual points indicate (**b**) delta or (**c**) absolute values for each subject. Significance level at *q* = 0.05, false discovery rate (FSD) corrected for multiple comparisons. ns: non-significant.

**Figure 5:**
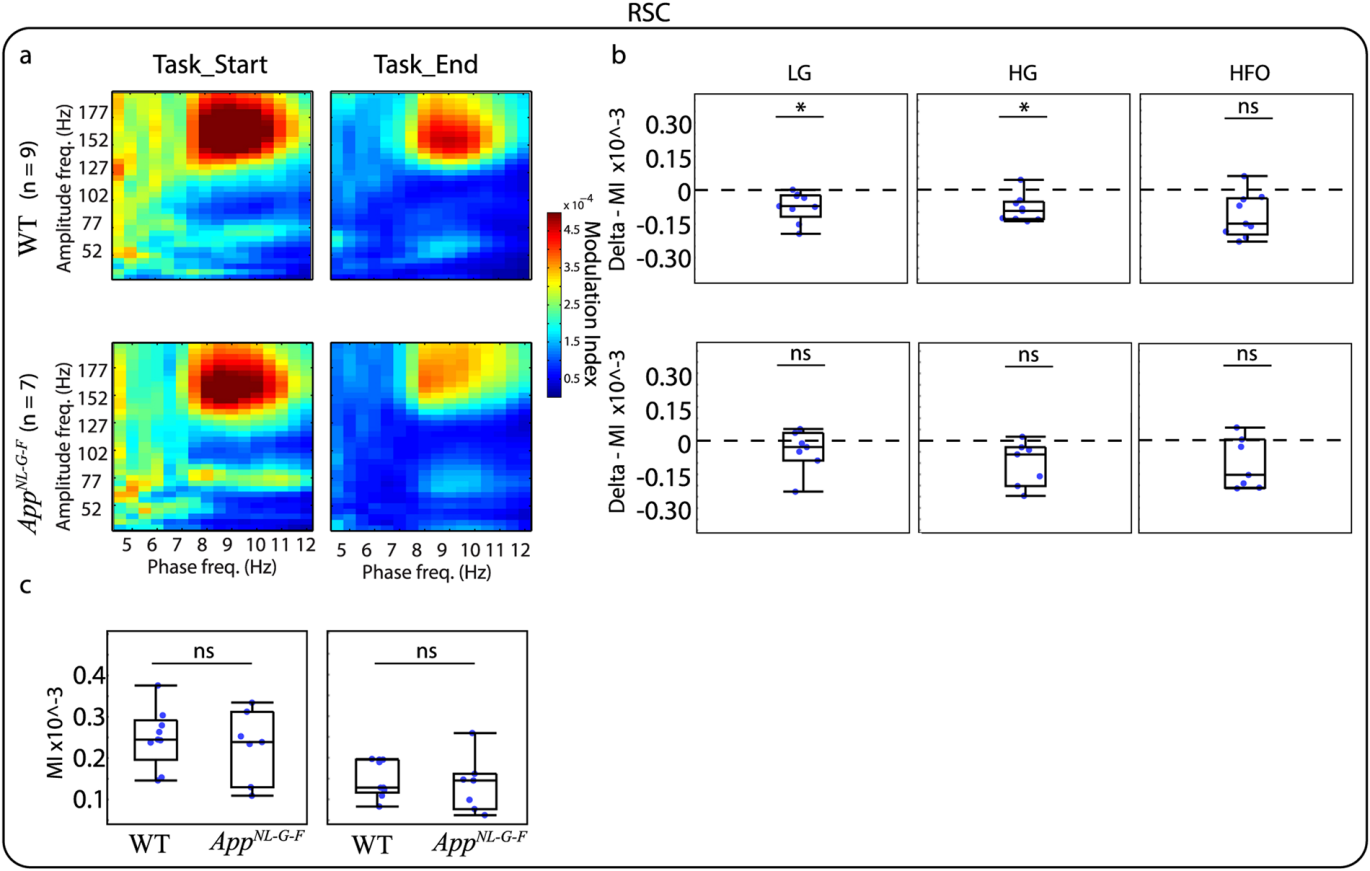
Mean phase-amplitude coupling (PAC) during the visual discrimination (VD) task for the retrosplenial cortex (RSC). (**a**) Phase-amplitude comodulograms plotted for WT mice (top panels) and *App*^*NL-G-F*^ mice (lower panels) for start of the VD task (Task_Start, left) and end of the VD task (Task_End, right). (**b**) Modulation index (MI) delta between Task_End and Task_Start for low gamma (LG, left), high gamma (HG, middle) and high frequency oscillations (HFO, right) for WT mice (top panels) and *App*^*NL-G-F*^ mice (lower panels). Comparison at each frequency band showed a significant different for WT mice at LG and HG, but no significant difference for *App*^*NL-G-F*^ mice. (**c**) MI between WT and *App*^*NL-G-F*^ mice for Task_Start (left) and Task_End (right). Comparison at each frequency band showed no significant difference. Values are represented in box plots were individual points indicate (**b**) delta or (**c**) absolute values for each subject. Significance level at *q* = 0.05, false discovery rate (FSD) corrected for multiple comparisons. ns: non-significant.

**Figure 6:**
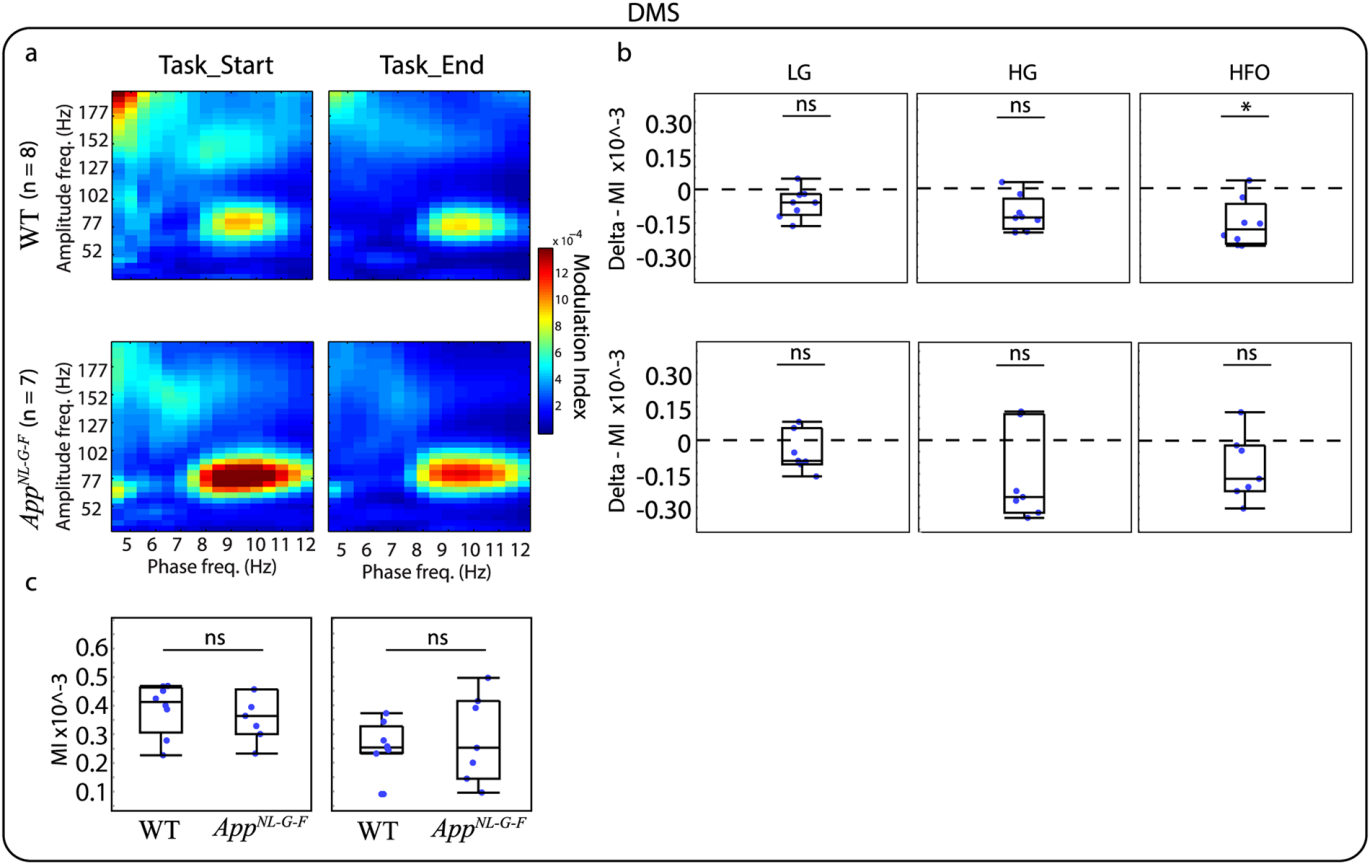
Mean phase-amplitude coupling (PAC) during the visual discrimination (VD) task for the dorsal medial striatum (DMS). (**a**) Phase-amplitude comodulograms plotted for WT mice (top panels) and *App*^*NL-G-F*^ mice (lower panels) for start of the VD task (Task_Start, left) and end of the VD task (Task_End, right). (**b**) Modulation index (MI) delta between Task_End and Task_Start for low gamma (LG, left), high gamma (HG, middle) and high frequency oscillations (HFO, right) for WT mice (top panels) and *App*^*NL-G-F*^ mice (lower panels). Comparison at each frequency band showed a significant different for WT mice at HFO, but no significant difference for *App*^*NL-G-F*^ mice. (**c**) MI between WT and *App*^*NL-G-F*^ mice for Task_Start (left) and Task_End (right). Comparison at each frequency band showed no significant difference. Values are represented in box plots were individual points indicate (**b**) delta or (**c**) absolute values for each subject. Significance level at *q* = 0.05, false discovery rate (FSD) corrected for multiple comparisons. ns: non-significant.

**Figure 7:**
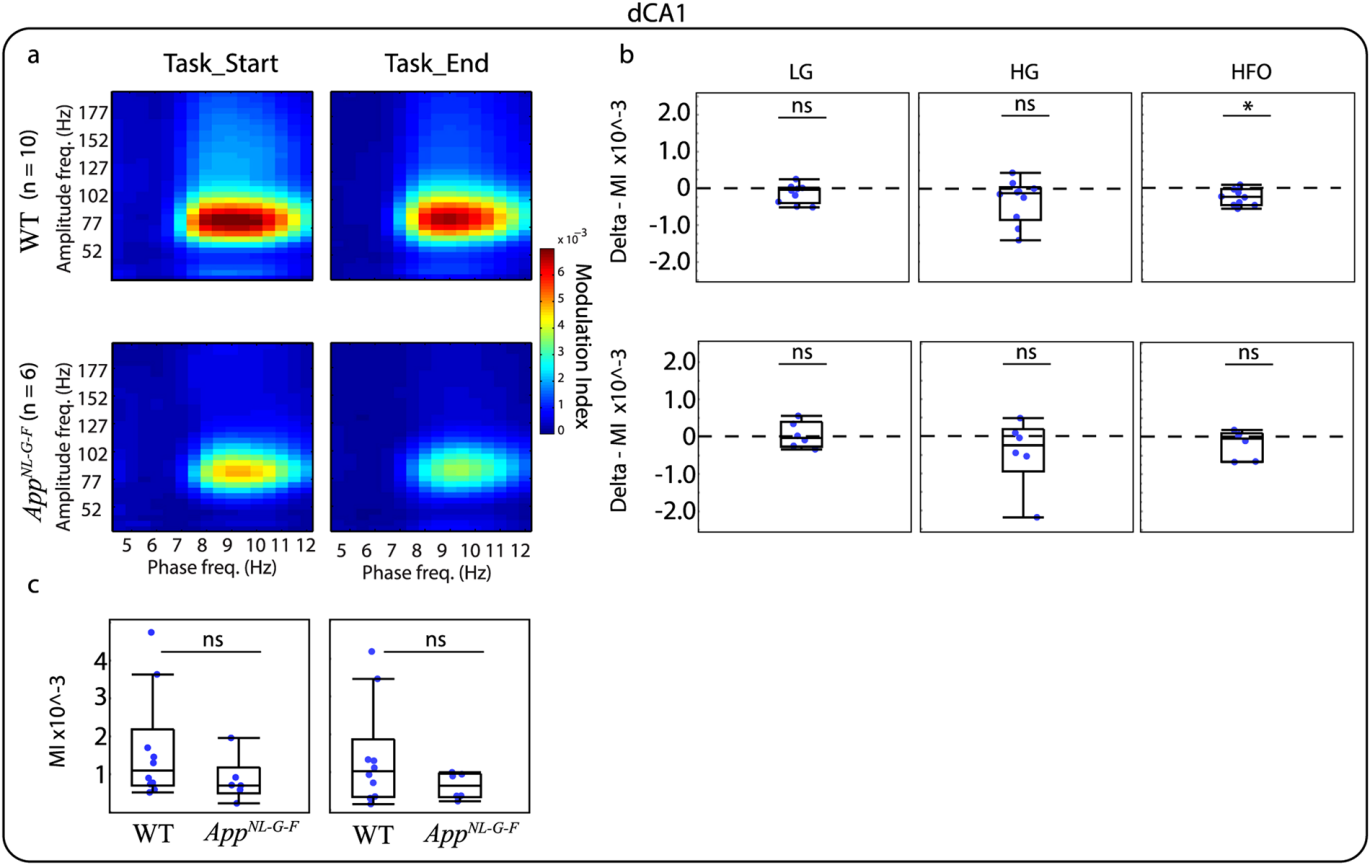
Mean phase-amplitude coupling (PAC) during the visual discrimination (VD) task for the dorsal CA1 region of the hippocampus (dCA1). (**a**) Phase-amplitude comodulograms plotted for WT mice (top panels) and *App*^*NL-G-F*^ mice (lower panels) for start of the VD task (Task_Start, left) and end of the VD task (Task_End, right). (**b**) Modulation index (MI) delta between Task_End and Task_Start for low gamma (LG, left), high gamma (HG, middle) and high frequency oscillations (HFO, right) for WT mice (top panels) and *App*^*NL-G-F*^ mice (lower panels). Comparison at each frequency band showed a significant different for WT mice at HFO, but no significant difference for *App*^*NL-G-F*^ mice. (**c**) MI between WT and *App*^*NL-G-F*^ mice for Task_Start (left) and Task_End (right). Comparison at each frequency band showed no significant difference. Values are represented in box plots were individual points indicate (**b**) delta or (**c**) absolute values for each subject. Significance level at *q* = 0.05, false discovery rate (FSD) corrected for multiple comparisons. ns: non-significant.

Similarly to relative PSD analysis, we investigated whether the modulation index (MI) changed as the animals progressed on the VD task and if this modulation was different for the *App*^*NL-G-F*^ compared to the WT mice. For this analysis, we grouped amplitude frequencies in three bands: Low Gamma (LG, 30–60 Hz), High Gamma (HG, 61–100 Hz), and HFO (101–200 Hz). This banding was selected as it has been suggested that different frequency bands act as different channels for communication. For instance, in the CA1 region of the hippocampus, gamma oscillations split into two different components indicating different origins. Low-gamma arises from interactions with the CA3 region of the hippocampus, while high-gamma is thought to arise from interactions with the entorhinal cortex^30^. A visual inspection of the phase-amplitude comodulograms of the different brain areas indicates that PAC occurs in different frequency bands. For instance, Cg (Fig. 4a) and RSC (Fig. 5a) show coupling between HFO and higher theta, while the DMS (Fig. 6a) and dCA1 (Fig. 7a) show coupling between HG and high theta. When investigating the changes in the MI between the end and the start of the VD task the most prominent effect was observed in the Cg (Fig. 4b) and RSC (Fig. 5b) for WT mice where the MI decreased by the end of the VD. WT mice also demonstrated a similar effect in HFO for the DMS (Fig. 6b) and dCA1 (Fig. 7b). Importantly, during the start of the task, the coupling seems to be associated with more frequency bands, suggesting that as the mice learned the task, the coupling became more localized. These differences were not clearly observed for the *App*^*NL-G-F*^ mice, where they only demonstrated a decreased coupling for HG in the Cg (Fig. 4b). See Table 2, for completed descriptive statistics and statistical analysis. Next, we investigated genotype differences within each condition. For this, we compared the MI for the three different amplitude bands at the start and the end of the task and corrected for multiple comparisons using a false discovery rate (FDR), as done with all previous comparisons (see “Methods” section). Table 3 shows that no significant differences were observed between *App*^*NL-G-F*^ and WT mice for any of the brain areas and frequency bands. Note that for simplicity, panel c of Figs 4–7 shows box plots of the full amplitude range, but the statistical analysis was done for each separate frequency band. It should be noted that some of the mean phase-amplitude comodulograms might visually suggest changes between genotypes, but as it can be seen in the box plots, some of these measurements had a high level of variability. The different results obtained for the within and between-subject analysis might reflect difference in the experimental design. Importantly, these designs are addressing different questions, therefore, they may provide different patterns of results^31^.

**Table 2:** Phase-amplitude coupling (PAC) during the visual discrimination (VD) task.

Genotype	Brain Area	Freq	Condition	Mean	SD	Median	Range	N	Z	Prob > [Z]	p value rank	FDR threshold
WT	Cg	LG	Task_Start	0.000136	0.000054	0.000133	0.000150	8	**−17**	**0.0156**	**7**	**0.0145833**
			Task_End	0.000060	0.000020	0.000066	0.000048					
		HG	Task_Start	0.000167	0.000056	0.000164	0.000201		**−17**	**0.0156**	**6**	**0.0125**
			Task_End	0.000072	0.000034	0.000065	0.000094					
		HFO	Task_Start	0.000271	0.000060	0.000283	0.000193		**−18**	**0.0078**	**1**	**0.0020833**
			Task_End	0.000138	0.000051	0.000143	0.000172					
*App* ^*NL-G-F*^		LG	Task_Start	0.000119	0.000074	0.000124	0.000214	7	−8	0.2188	20	0.0416667
			Task_End	0.000073	0.000035	0.000085	0.000084					
		HG	Task_Start	0.000219	0.000173	0.000181	0.000506		−11	0.0781	15	0.03125
			Task_End	0.000099	0.000040	0.000098	0.000120					
		HFO	Task_Start	0.000247	0.000090	0.000229	0.000249		**−14**	**0.0156**	**8**	**0.0166667**
			Task_End	0.000135	0.000065	0.000099	0.000174					
WT	dCA1	LG	Task_Start	0.000852	0.000477	0.000713	0.001435	10	−13.5	0.1934	19	0.0395833
			Task_End	0.000719	0.000499	0.000732	0.001614					
		HG	Task_Start	0.003142	0.003040	0.001842	0.002389		−14.5	0.1602	18	0.0375
			Task_End	0.002817	0.003087	0.001674	0.008305					
		HFO	Task_Start	0.001266	0.001129	0.000807	0.003486		**−24.5**	**0.0098**	**4**	**0.0083333**
			Task_End	0.001019	0.001000	0.000786	0.003212					
*App* ^*NL-G-F*^		LG	Task_Start	0.000332	0.000204	0.000310	0.000581	6	1.5	0.8438	24	0.05
			Task_End	0.000371	0.000320	0.000257	0.000816					
		HG	Task_Start	0.001877	0.001494	0.001523	0.004300		−3.5	0.5625	23	0.0479167
			Task_End	0.001438	0.008713	0.001322	0.002162					
		HFO	Task_Start	0.000597	0.000397	0.000438	0.001036		−4.5	0.4375	22	0.0458333
			Task_End	0.000398	0.000182	0.000377	0.000465					
WT	DMS	LG	Task_Start	0.000176	0.000071	0.000156	0.000201	8	−15	0.0391	12	0.025
			Task_End	0.000115	0.000036	0.000119	0.000092					
		HG	Task_Start	0.000434	0.000149	0.000443	0.000412		−16	0.0234	10	0.0208333
			Task_End	0.000322	0.000130	0.000318	0.000340					
		HFO	Task_Start	0.000434	0.000112	0.000433	0.000326		**−17**	**0.0156**	**5**	**0.0104167**
			Task_End	0.000276	0.000121	0.000296	0.000390					
*App* ^*NL-G-F*^		LG	Task_Start	0.000190	0.000074	0.000205	0.000205	7	−9	0.1563	17	0.0354167
			Task_End	0.000136	0.000047	0.000158	0.000120					
		HG	Task_Start	0.000703	0.000571	0.000494	0.001642		−11	0.0781	14	0.0291667
			Task_End	0.000535	0.000505	0.000267	0.001350					
		HFO	Task_Start	0.000350	0.000098	0.000360	0.000246		−11	0.0781	13	0.0270833
			Task_End	0.000230	0.000154	0.000188	0.000480					
WT	RSC	LG	Task_Start	0.000147	0.000071	0.000119	0.000218	9	−19.5	0.0195	9	0.01875
			Task_End	0.000075	0.000023	0.000075	0.000067					
		HG	Task_Start	0.000177	0.000053	0.000181	0.000189		**−21.5**	**0.0078**	**3**	**0.00625**
			Task_End	0.000091	0.000034	0.000087	0.000095					
		HFO	Task_Start	0.000309	0.000097	0.000326	0.000332		**−21.5**	**0.0078**	**2**	**0.0041667**
			Task_End	0.000196	0.000068	0.000182	0.000178					
*App* ^*NL-G-F*^		LG	Task_Start	0.000130	0.000091	0.000089	0.000268	7	−6	0.375	21	0.04375
			Task_End	0.000084	0.000043	0.000084	0.000129					
		HG	Task_Start	0.000202	0.000118	0.000188	0.000336		−10	0.1094	16	0.0333333
			Task_End	0.000098	0.000044	0.000089	0.000120					
		HFO	Task_Start	0.000272	0.000089	0.000302	0.000225		−13	0.0313	11	0.0229167
			Task_End	0.000166	0.000096	0.000155	0.000289					

Brain areas: Cingulate Cortex (Cg), dorsal CA1 region of the hippocampus (dCA1), dorsal medial striatum (DMS), and retrosplenial cortex (RSC).

Frequencies: theta, gamma and high frequency oscillations (HFO).

Conditions: Task_Start = start of the VD task, Task_End = end of the VD task.

Descriptive statistics for Modulation Index (MI), N: sample size, Z: Wilcoxon rank-sum test.

Significance level at *q* = 0.05, false discovery rate (FSD) corrected for multiple comparisons.

Bold cells indicate statistically significant values. P value ranks lower than 8 are statistically significant different between the two conditions.

**Table 3:** Between-subject analysis of phase-amplitude coupling (PAC) during the visual discrimination (VD) task.

Condition	Brain Area	Freq	Z	Prob > [Z]	p value rank	FDR threshold
Task_Start	Cg	LG	−0.6365	0.5244	14	0.0292
		HG	0.28932	0.7723	20	0.0417
		HFO	−0.6365	0.5244	15	0.0313
	dCA1	LG	−2.22354	0.0262	1	0.0021
		HG	−0.27116	0.7863	21	0.0438
		HFO	−1.57275	0.1158	2	0.0042
	DMS	LG	0.17359	0.8622	23	0.0479
		HG	0.98368	0.3253	9	0.0188
		HFO	−1.33087	0.1832	5	0.0104
	RSC	LG	−0.74096	0.4587	12	0.0250
		HG	0.10585	0.9157	24	0.0500
		HFO	−0.74096	0.4587	13	0.0271
Task_End	Cg	LG	0.86796	0.3854	11	0.0229
		HG	1.56232	0.1182	3	0.0063
		HFO	−0.6365	0.5244	16	0.0333
	dCA1	LG	−1.24735	0.2123	6	0.0125
		HG	−0.59656	0.5508	17	0.0354
		HFO	−1.24735	0.2123	7	0.0146
	DMS	LG	1.33087	0.1832	4	0.0083
		HG	0.40505	0.6854	19	0.0396
		HFO	−1.09941	0.2716	8	0.0167
	RSC	LG	0.2117	0.8323	22	0.0458
		HG	0.4234	0.672	18	0.0375
		HFO	−0.95266	0.3408	10	0.0208

Conditions: Task_Start = start of the VD task, Task_End = end of the VD task.

Brain areas: Cingulate Cortex (Cg), dorsal CA1 region of the hippocampus (dCA1), dorsal medial striatum (DMS), and retrosplenial cortex (RSC).

Frequencies: low gamma (LG), high gamma (HG), and high frequency oscillations (HFO).

Z: Wilcoxon rank-sum test.

Significance level at *q* = 0.05, false discovery rate (FSD) corrected for multiple comparisons.

### Brain oscillations analysis during home environment exploration

#### Relative PSD

Our next analysis was conducted to determine if we could observe different brain oscillatory activity when animals were exploring the home environment at an age of 5 months compared to 8 months. As with the relative PSD during the task, we did not observe difference for any of the frequency bands between genotypes (Table 4). Furthermore, age did not have an effect of the PSD for either the *App*^*NL-G-F*^ and WT mice (Supplementary Table 2).

**Table 4:** Between-subject analysis of relative power spectral density (PSD) during the no-task condition.

Condition	Brain Area	Freq	Z	Prob > [Z]	p value rank	FDR threshold
5_Months	Cg	Theta	0.6365	0.5244	14	0.0291667
		Gamma	−0.05786	0.9539	24	0.05
		HFO	−0.40505	0.6854	19	0.0395833
	dCA1	Theta	0.88388	0.3768	9	0.01875
		Gamma	0.29463	0.7683	20	0.0416667
		HFO	0.76603	0.4437	11	0.0229167
	DMS	Theta	0.63888	0.5229	13	0.0270833
		Gamma	−0.5111	0.6093	17	0.0354167
		HFO	−0.7665	0.4433	10	0.0208333
	RSC	Theta	0.28932	0.7723	21	0.04375
		Gamma	−0.40505	0.6854	18	0.0375
		HFO	−0.52077	0.6025	16	0.0333333
8_Months	Cg	Theta	−0.96825	0.3329	7	0.0145833
		Gamma	0.71005	0.4777	12	0.025
		HFO	1.74284	0.0814	2	0.0041667
	dCA1	Theta	−0.13333	0.8415	23	0.0479167
		Gamma	0.26667	0.7897	22	0.0458333
		HFO	1.2	0.2301	5	0.0104167
	DMS	Theta	−1.5	0.1336	3	0.00625
		Gamma	2.07143	0.0383	1	0.0020833
		HFO	0.92857	0.3531	8	0.0166667
	RSC	Theta	1.09735	0.2725	6	0.0125
		Gamma	−0.58095	0.5613	15	0.03125
		HFO	−1.22644	0.22	4	0.0083333

Conditions: 5 and 8 months of age.

Brain areas: Cingulate Cortex (Cg), dorsal CA1 region of the hippocampus (dCA1), dorsal medial striatum (DMS), and retrosplenial cortex (RSC).

Frequencies: theta, gamma, and high frequency oscillations (HFO).

Z: Wilcoxon rank-sum test.

Significance level at *q* = 0.05, false discovery rate (FSD) corrected for multiple comparisons.

#### Phase-amplitude coupling (PAC)

As a with the relative PSD, no significant differences were observed between genotypes during the home environment exploration (Table 5, Supplementary Figures 1, 2, 3, and 4) for any of the brain areas. Furthermore, we did not observe an effect on age for either the WT and *App*^*NL-G-F*^ mice (Supplementary Table 3). Taken together, these results suggest that the *App*^*NL-G-F*^ mice demonstrate up to 8 months of age normal PAC in a freely moving condition without task performance (investigation of further age points were beyond the scope of this study).

**Table 5:** Between-subject analysis of phase-amplitude coupling (PAC) during the no-task condition.

Condition	Brain Area	Freq	Z	Prob > [Z]	p value rank	FDR threshold
5_Months	Cg	LG	−0.68264	0.4948	12	0.02609
		HG	1.31276	0.1893	6	0.01304
		HFO	−0.26255	0.7929	22	0.04783
	dCA1	LG	−1.37607	0.1688	3	0.00652
		HG	−0.52926	0.5966	14	0.03043
		HFO	−0.31755	0.7508	19	0.04130
	DMS	LG	1.44659	0.148	1	0.00217
		HG	0.52077	0.6025	15	0.03261
		HFO	0.28932	0.7723	21	0.04565
	RSC	LG	0.36757	0.7132	18	0.03913
		HG	1.20774	0.2271	7	0.01522
		HFO	−0.15753	0.8748	24	0.05217
8_Months	Cg	LG	−0.40505	0.6854	16	0.03478
		HG	−0.40505	0.6854	17	0.03696
		HFO	1.33087	0.1832	5	0.01087
	dCA1	LG	−1.35529	0.1753	4	0.00870
		HG	−0.88388	0.3768	10	0.02174
		HFO	−0.53033	0.5959	13	0.02826
	DMS	LG	0.7665	0.4433	11	0.02391
		HG	1.14998	0.2502	8	0.01739
		HFO	0.89443	0.3711	9	0.01957
	RSC	LG	−0.28932	0.7723	20	0.04348
		HG	−0.17359	0.8622	23	0.05000
		HFO	1.44659	0.148	2	0.00435

Conditions: 5 and 8 months of age.

Brain areas: Cingulate Cortex (Cg), dorsal CA1 region of the hippocampus (dCA1), dorsal medial striatum (DMS), and retrosplenial cortex (RSC).

Frequencies: low gamma (LG), high gamma (HG), and high frequency oscillations (HFO).

Z: Wilcoxon rank-sum test.

Significance level at *q* = 0.05, false discovery rate (FSD) corrected for multiple comparisons.

### Pathology

**Figure 8:**
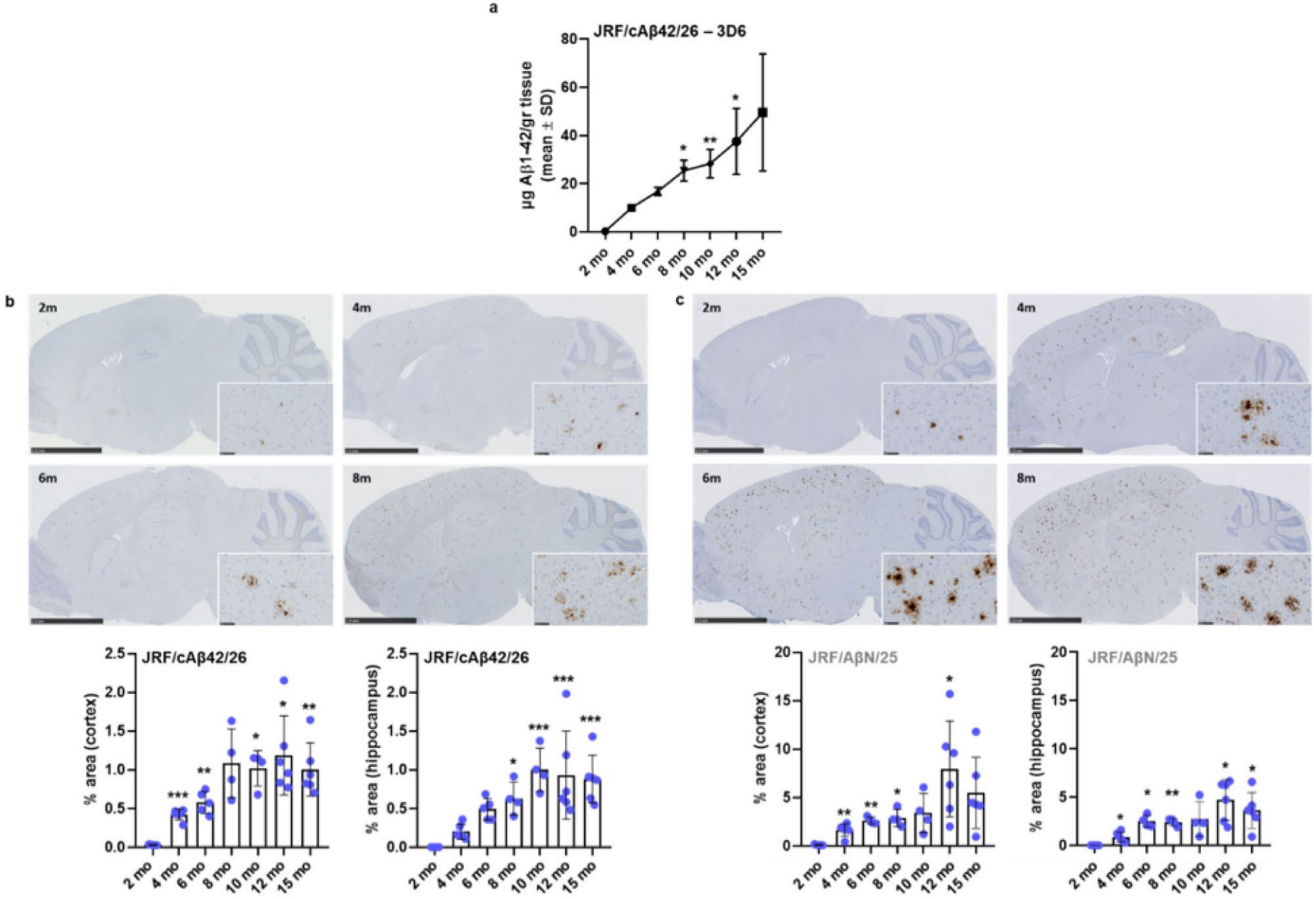
Amyloid β pathology in *App*^*NL-G-F*^ mice. (**a**) Biochemical quantification of Aβ1–42 in *App*^*NL-G-F*^ *fore*brains (GuHCl fraction) measured by sandwich ELISA. Standard curves for calibration were generated using synthetic human Aβ1–42 peptide. Data represent the mean of 2 to 4 independent measurements of pooled samples with each pool consisting of equal volumes of extracts from 4 to 6 mice (n = 5, 6, 5, 4, 4, 6, 6 per indicated time point respectively). Star symbols indicate significance compared to 2 mo (months of age). (**b**,**c**) Example images and immunohistochemical analysis of the plaque occupancy area in cortex (inset) and hippocampus using a C-terminal antibody (JRF/cAβ42/26, b) or N-terminal antibody (JRF/AβN/25. (**c**) Scale bars in the photographs are 2.5 mm and 50 µm (inset). In the summarizing plots, mean and SD are presented in a bar chart in which each superimposed dot representing a single animal. Star symbols indicate significance compared to 2 mo.

Biochemical analysis of *App*^*NL-G-F*^ forebrain tissue revealed that Aβ1–42 increased in an age-dependent manner (Fig. 8a) and was accompanied by progressive Aβ plaque pathology as seen by immunohistochemistry (Fig. 8b,c). Aβ1–42 levels in the brain seemed to be minor at 2 months of age, but progressively increased starting from 4 months of age up to 12–15 months. This can also be deduced from the immunohistochemical evaluation of cortical and hippocampal pathology (% Aβ plaque area as detected by antibodies JRF/cAβ42/26 and JRF/AβN/25) that showed a limited number of Aβ deposits at 2 months of age in cortex, further progressing up to 8–15 months as observed by others^19,21,32^. Altogether, our data indicate that between 4 and 8 months of age, the period where mice were used for the *in vivo* behavioural and neurophysiological characterisation, increased Aβ1–42 levels and Aβ plaques were present in both cortex and hippocampus.

## Discussion

The purpose of this study was to characterize *App*^*NL-G-F*^ mice, a second-generation mouse model of Aβ amyloidosis^19^, by performing behavioral, electrophysiological, and pathological investigations in an age-dependent manner. Previous research using APP-overexpressing models (first-generation mouse models) has led to the hypothesis that aberrant network and altered oscillatory rhythmic activity caused by Aβ amyloidosis reflect and underlie the early cognitive disturbances observed in AD^13,16,33^. In our study, we tested this hypothesis using a more relevant model of AD pathology, as the *App*^*NL-G-F*^ mice produce robust Aβ amyloidosis, like the first-generation models^34^ and AD patients^35^, but without potential undesirable side effects caused by the overexpression of APP^9,10^.

Behaviorally, the *App*^*NL-G-F*^ mice did not show strong associative learning impairments in the VD task. Overall *App*^*NL-G-F*^ mice demonstrated a higher variability than the controls in learning the task. Mice were 4.5 months old at the start of the VD task, which correlates with early plaque deposition. Previous studies characterizing the new-generation of AD mouse models have identified some minimal behavioral changes at younger ages^23^, though impaired performance on cognitive tests has only been reported from the age of six months onwards^19,21,22^. Our study, using a different behavioral task than those already investigated in this model, contributes to an increasing body of evidence indicating that the *App*^*NL-G-F*^ mice do not show severe cognitive impairments at early plaque stages. On the other hand, APP-overexpressing models show more variability with respect to the onset of cognitive deficits in relation to pathology^8,36^.

To investigate the electrophysiological correlates, we used advanced techniques to explore the effects of AD-related pathology on neural oscillations in different brain areas during cognition. Electrode placement targeting different brain areas was motivated by two main factors: topographic distribution of amyloid plaques in AD patients^37^ and animal models with Aβ pathology^7^, and the functional role of these areas on cognitive processes associated with the VD task, especially for the DMS, which plays a role in establishing action-reward associations^38^. We focussed our analysis on PAC, as this measurement has been suggested to be associated with normal cognitive functioning in humans^26,39,40^ and rodents^27,41–43^, and to be disrupted in patients with AD and MCI^15,44^. Our analysis was centered on comparing the start and end of the VD task. At the end of the task, the relationship between decision making and coupling could be easily assessed, as mice were at 80% or higher accuracy. For the start of the task, the interpretation was more difficult, as mice were responding at chance level; therefore, some of the correct trial responses analysis could include brain activity that may or may not pertain to decision making. We believe that some of the non-specific coupling observed at the start of the task could be associated with this mentioned variability within correct responses. Our results indicate a decrease in coupling for the WT mice as they learned the task, especially for the Cg and RSC. This result is in accordance with previous research indicating that PAC increases with task difficulty^26,45^. For the *App*^*NL-G-F*^ mice, this decrease was less pronounced, only reaching statistical significance for HG in the Cg. Importantly, between-subject comparisons did not reveal significant differences between the two genotypes. Notable there was no effect on power for any of the tested comparisons. On the other hand, multiple studies using different transgenic rodent models including human APP-overexpression transgenes with different familial AD mutations have demonstrated gamma power alterations^46–48^ as well as PAC dysfunctions^14,48^ before major amyloid plaque accumulation. Furthermore, studies, such as the ones carried out by Poza and colleagues^15^ or Dimitriadis and colleagues^44^ provide valuable insights to better understand PAC changes associated with the disease, but both studies reported PAC alterations in AD and MCI patients respectively. Considering the difficulty to recruit patients in the preclinical phase, relevant animal models could help to further understand the potential use of PAC measurement as a biomarker for AD. The *App*^*NL-G-F*^ mice at the ages investigated in our study demonstrate Aβ accumulation without clear cognitive symptoms, making them a relevant preclinical AD model. Altogether our results indicate very subtle changes in coupling, suggesting PAC might not be affected in the preclinical phase of AD based on amyloidosis pathology only. It remains to be tested if other brain areas or the interaction between them might reveal changes in PAC at these early stages. Furthermore, other complex functional connectivity analysis that has been explored in patients^49^, should be investigated in this and other animal models of AD pathology.

Our main motivation for this study was to investigate PAC, for which a role in neuronal information processing was reported^40^, in a second-generation mouse model while animals performed the task. In addition, we were also interested in investigating PAC without any influence that the VD task could have on this readout. To this end, we measured LFPs when mice were exploring their home environment at 5 and 8 months of age. Despite seeing an age-dependent increase in Aβ pathology between these time-points, we did not observe changes in PAC for any of the brain regions. Importantly, the ages for recording were selected based on previous reports of pathology^19^. Our objective was to obtain two measurements: one at a relatively early plaque stage and a second one at a later time-point where amyloid pathology is at an advanced state. Our findings indicate an age-dependent increase in Aβ1–42 levels and plaque deposition that are consistent with previous research^19,21,32^. Previous reports indicated the absence of hyperexcitability in the *App*^*NL-G-F*^ at 8 and 12 months of age^24^ and subtle synaptic alterations at around 18 months^50^. However, other reports have indicated theta-gamma coupling impairments in the entorhinal cortex of *App*^*NL-G-F*^ mice at 5 months of age^51^ and hypersynchronous functional connectivity of brain networks using resting-state functional MRI in *App*^*NL-G-F*^ mice at 3 months (pre-plaque stage)^52^. Taken together, these studies suggest that there is evidence for disruptions in network function under certain circumstances, but these are not apparent with all methodologies. Further work is needed to better understand how the variability in the methods and measurements of these studies leads to different outcomes about different aspects of network integrity in these animals. Finally, an important consideration for the translatability of findings in second-generation models is that while they avoid potential artefacts associated with the overexpression of APP, the development of pathology relies on several mutations linked to familial forms of AD, and may not accurately recreate the pathological processes in sporadic forms of AD. Furthermore, like the first-generation models, the *App*^*NL-G-F*^ mice do not exhibit tau pathology or neurodegeneration, which are important components of AD pathology.

Altogether, our results do not support the hypothesis of early alterations in oscillations and functional neuronal activity^13,14,33^ postulated using the first-generation models. We believe that by allowing researchers to dissociate the effects of APP overexpression from the pathophysiological changes in AD, the second-generation models will facilitate a deeper understanding of the neurobiology of the disease. In AD patients changes in electroencephalography (EEG) power spectral or synchronization have been commonly reported^3,49,53,54^. Although some of these studies used new guidelines for diagnosis and investigated preclinical (before cognitive symptoms) and clinical AD; the reliability of the AD diagnosis in other studies must be considered with caution, given the lack of available biomarkers at the time of the studies. For instance, it has been indicated that the discrepancy between clinical and pathological diagnosis is about 20%^55^. Many studies have also shown that AD patients have a higher risk of developing seizures and epilepsy^56–58^; however, the reported prevalence in the literature fluctuates from 1.5 to 64%^5^. Furthermore, it is still unclear whether neuronal alterations occur rather at a late stage of the disease as a consequence of neurodegeneration, or at an early stage as a primary mechanism contributing to cognitive dysfunction. If the second conjecture is correct, the potential value of having a reliable biomarker of preclinical and early stages of the AD could have a major impact on the disease diagnostics. Our knowledge about the pathophysiology of neural networks in AD is limited and more research is urgently needed. In parallel to research carried-out with patients, studies investigating electrophysiological readouts of the *App*^*NL-G-F*^ model could also help to gain new insights and outline a timeline on network changes associated with AD pathology.

## Methods

### *In Vivo* Experiments Methods

#### Animals

Data were obtained from eight homozygous male *App*^*NL-G-F*^ mice (generated by Saito and colleagues^19^ and obtained from the Janssen transgenic rodent facility, Belgium) and ten age-matched non-litter mates WT C57BL/6J male mice (Charles River, France). Mice were singly housed in individually ventilated cages under a reversed 12–12 light cycle (lights off 07:00–19:00; light intensity ~100 lux) under controlled environmental conditions throughout the study (22 ± 2 °C ambient temperature and relative humidity at 60%). Home cages were equipped with corn cob bedding, a tinted polycarbonate shelter and tissue for nesting material and *ad libitum* water. Throughout pre-training and cognitive testing, mice were provided with a restricted diet (Dustless precision pellets, Bioserv, USA) to maintain them at 80% free-fed weight to ensure consistent motivation towards reward pellets. All behavioral testing was conducted during the dark phase to obtain optimal engagement in the behavioral task as this is the active phase of the circadian cycle in mice.

All *in vivo* and *in vitro* studies were performed in strict accordance with the guidelines of the Association for Assessment and Accreditation of Laboratory Animal Care International (AAALAC) and with the European Council Directive of 24 November 1986 (86/609/EEC) and European Ethics Committee directive (2010/63/EU) for the protection of laboratory animals. In line with Belgian governmental directives all protocols were approved by the Animal Care and Use Committee of Janssen Pharmaceutica NV.

#### Surgery

Surgeries were carried out when mice were three months old. The surgeon was blinded to animal genotype before carrying out the procedure. Anesthesia was induced via two minutes isoflurane inhalation (O_2_, N_2_O and 5% isoflurane) and animals inserted into a stereotactic frame (StereoDrive, Neurostar, Germany). During the surgical procedure, anesthesia was maintained using a continuous flow of gas (O_2_, N_2_O and 2% isoflurane) delivered via inhalation mask. A homeothermic blanket system was used to sustain a stable 37–38 °C body temperature. A subcutaneous injection of analgesic Piritramide (dipidolor, 0.025 mg/kg) was administered. As a further precaution to minimize pain from the surgery, a local spray analgesic (Xylocaine, 10%) was applied at the surgery site. An incision was made along the sagittal plane to expose the skull, and the scalp held open using suture thread tied to the stereotactic frame. The skull was cleaned using saline solution and dried using swabs and cotton buds. Holes were drilled for the placement of four recording electrodes; all coordinates relative to bregma, anterior-posterior (AP), medial-lateral (ML), dorsal-ventral (DV)^59^. Two stainless steel screws were affixed over the left frontal and right occipital lobes to secure the implant. Depth electrodes consisted of a single fomvar-insulated tungsten wire (100 μm diameter with a blunt-tipped, Peira, Belgium) and were inserted into the DMS (1.4 mm AP, −1 mm ML, 2.5 mm DV), and the dCA1 (−1.7 mm AP, −1.5 mm ML, 1.7 mm DV). Surface electrodes (500 μm diameter gold-plated pins, Z = 150) were placed in the Cg (−0.5 mm AP, 0.0 mm ML) and the RSC (−1.8 mm AP, 0.0 mm ML). All electrodes were referenced to the same ground electrode placed on the midline over the cerebellum (−1.5 mm AP, 0.5 mm ML) and grounded by a pin positioned on the midline over the occipital lobe (−5.0 mm AP, 0.0 mm ML). After placing all electrodes, a multichannel connector (Nano strip connector, Omnetics, Minneapolis, USA) was affixed using dental cement to the cranium and the wound sutured around the implant. Mice’s recovery and well-being was closely monitored until they were fully recovered (approximately ten days).

### Visual discrimination (VD) Task

#### Apparatus

Mice were tested in customized operant chambers (modified from Med Associates Inc. Fairfax, Vermont): two sides of the box were constructed of clear Perspex. One of the other two sides was equipped with a pellet receptacle containing a light and an infra-red nose-poke detector, a tone generator, and a house light. The remaining side of the box was equipped with a touch-sensitive, flat-screen, LCD computer monitor. Nose-poking on the screen was detected with an infrared touch detection system. The monitor was then covered with a “mask”, a piece of black Perspex with two aperture plate dividing the touchscreen into two response fields (75 mm × 75 mm) in which visual stimuli were presented. Boxes were placed in sound attenuated chambers fitted with a small ventilation fan that also provided a mild masking background noise. The floor of the chamber consisted of aluminum bars spaced approximately 1 cm apart. Each operant box was controlled by K-limbic software, version 1.20.2 (Conclusive Solutions, Sawbridgeworth, UK).

### Touchscreen pre-training stages

The shaping of animals to use touch screens to respond to stimulus images consisted of five pre-training stages^25^. All mice began pretraining at 3.5 months of age and progressed from each stage on an individual basis based on their performance (i.e. reaching a pre-set performance criterion).

#### Habituation

The first stage aimed to familiarize the mice with the operant chambers and extractor fan noise. Mice were placed in the boxes, lights off, for 30 minutes and given 10 reward pellets (TestDiet, USA). Mice were required to consume all reward pellets during the session to progress to the next stage.

#### Tone association

In this stage mice established an association between a tone and reward delivery. A reward was delivered with a tone to begin the session. The food magazine light remained on from reward delivery until collection. The next reward and tone were delivered after a 30 second inter-trial interval (ITI) when the mouse entered the food magazine. Mice were required to complete 60 trials within 60 minutes for two consecutive days to advance to the next stage.

#### Touch association

Here, mice were encouraged to touch the screen to receive the reward. During trials, a white square was presented in one of the two response windows for 30 seconds. The location of the stimulus presentation was pseudo-random between trials. If mice touched the square during this time a food reward was delivered with a tone and a 10 second ITI initiated. Otherwise, the stimulus was removed, and the house light switched on for 10 seconds followed by a 10 second ITI. There was no penalty for touching the other response window. Mice were required to complete a minimum of 35 trials in 45 minutes to advance to the next stage.

#### Must touch

During this stage the association between screen touches and rewards was reinforced. The procedure and success criterion were the same as in the Touch Association stage, however new trials did not begin automatically after 30 seconds – animals were required to touch the illuminated square before the next trial could begin. Animals were required to complete a minimum of 35 trials in 45 minutes to advance to the next stage.

#### Punish incorrect

The final stage of pretraining introduced a penalty for indiscriminate screen touches. The trials proceeded as in the Must Touch stage but touches to the non-illuminated response window triggered a 5 second timeout with the house light on, followed by a 10 second ITI. These trials were recorded as incorrect and followed by a CT. During all CTs the stimulus presentation was in the same location as the preceding trial, and the trail repeated until the animal responded to the correct window. Outcomes during correction trials were not included in trial count or any analyses. Mice progressed from this stage once they could achieve ≥75% correct responses over a minimum of 30 trials for two consecutive days. The number of trials was capped at 80. During this stage of the pretraining regime mice were fitted with headstages used for the LFPs recordings on alternating test days to acclimatize them to wearing the headstage. After this step mice were ready to advance to the VD task testing.

### Visual discrimination (VD) task testing

VD testing began when mice were at an age of 4.5 months old. In VD sessions, a pair of images previously validated^25^ (Fig. 2a) were presented on screen. Based on a subject’s counterbalanced group assignment, each image was designated as either the *conditioned stimulus* (S+) or the *unconditioned stimulus* (S−) and the reward-contingency of the image was kept constant for each mouse across the experiment. Responses to S+ were rewarded with a food pellet, while responses to S− were recorded as incorrect and resulted in a 5 s timeout with the house light on. As in the *Punish Incorrect* pretraining stage, incorrect trials were followed by CTs in which stimulus locations were kept the same. Stimulus presentations occurred in a pseudo-random location in each trial, never appearing in the same window in more than three consecutive trials (excluding CTs) and total presentations counterbalanced between the two locations. Mice completed one 45-minute session (max 80 trials) of VD testing daily until they achieved an acquisition criterion set as two consecutive sessions at ≥80% correct responses over a minimum of 30 trials. For an illustration of the VD task see Fig. 2b.

### Local field potentials (LFPs) analysis

#### Recordings

LFPs were recorded with a sample frequency of 1000 Hz, high-pass filtered above 1 Hz, and referenced to the ground electrode placed midline above the cerebellum using small-size W4-HS wireless headstages weighing approximately 2.2 grams including battery, connected to a W2100 system (Multichannel systems, Germany. All analyses were done with built-in and custom-written routines in MATLAB (MathWorks, 2014a). Detection of artefacts and wrongly placed electrodes was carried out in three steps. Firstly, data were visually inspected and electrodes with noise were excluded for further analysis. Secondly, epochs were excluded during the Matlab analysis using an amplitude method (i.e. artefact rejection) with a standard deviation cut-off of 10. Finally, at the end of the experiment, mice were deeply anesthetized and electrolytical lesions were produced at the selected recording sites using a current generator apparatus (500 µA for 30 seconds, MC Stimulus II, Multichannel systems, Germany), then mice were euthanized, and their brain tissue was frozen in dry-ice cooled methylbutane. Coronal sections of frozen brains were obtained using a cryostat and counterstained for histological verification of subcortical electrode placements.

LFPs were recorded and analysed in two conditions:

- VD task: Neuronal and behavioral data were synchronized with a precision of 10 milliseconds using a transistor-transistor logic output signal generated by the food magazine light of the operant box. Recordings of the first (Task_Start) and last (Task_End) session of the VD task were included in the study. Response and collection latency data were used to select an epoch for analysis within each trial of 1.4 second prior to a correct touchscreen response (Fig. 2b). This value was used to maximize the length of the window containing brain activity related to choice-making immediately prior to screen touch while avoiding inclusion of activity related to trial initiation.
- Home-cage monitoring: Mice at 5 and 8 months of age were placed for 1 hour in their home-cage environment with a camera (UI-3140CP-C-HQ Rev.2, IDS Imaging, Germany) above the cage to record their activity for synchronization with recorded LFPs. Home-cages were placed in sound attenuated chambers fitted with a small ventilation fan that also provided a mild masking background noise. Behavioral activity was scored using Smart software v3.0 (Panlab, Barcelona) and categorized into different activity levels based on speed and only segments where the speed of the mice was between 2 and 30 cm/sec were considered for the analysis. Using a sliding window approach, we selected LFPs data at 1.4 seconds epochs for further analysis. The speed and epoch’s length parameters were selected to match behavioral task conditions.

### Relative power spectral density (PSD) analysis

Welch’s power spectral density was estimated and analyzed for frequencies ranging from 4 to 200 Hz for each epoch for the different conditions. PSD values were averaged in 1 Hz frequency bins across trials within a session for each subject and brain region and power was expressed as relative power to the total power over 4 to 200 Hz. For statistical analysis relative PSD data were averaged across theta (4–12 Hz), gamma (30–100 Hz) and HFO (101–200 Hz).

### Phase amplitude coupling (PAC)

The signal was convoluted with complex Morlet wavelets to extract estimates of time-varying frequency band-specific amplitude and phase from the LFPs data. Theta-gamma PAC was calculated using an algorithm described previously^27^. The MI was used to quantify the modulation of the high frequency amplitude signal (30–200 Hz, estimated in 5 Hz steps) by a low frequency phase signal (4–12 Hz, estimated in 0.5 Hz steps). While a MI value close to zero indicates no relationship between low frequency phase and high frequency amplitude, a higher value results from stronger phase-to-amplitude modulation. Due to the short duration of the analysis window (1.4 seconds), within each epoch a lengthiest segment with integer number of cycles for the considered low frequency phase was extracted, and values of analytical signals for all the segments within a session were aggregated (over time) in a complex phase space prior to MI estimations for each mice and electrode. For statistical analysis MI were averaged across the amplitude frequency low gamma (30–60 Hz), high gamma (61–100 Hz) and HFO (101–200 Hz).

### Statistics

Statistical analyses were conducted using JMP®, version 12 (SAS Institute Inc, NC, US). For behavioral testing, the distributions of dependent variables were assessed for meeting the assumption of normality required in parametric statistical tests. Group comparisons were made using a two-tailed two-sample *t*-test, or the normal approximation to the Wilcoxon rank-sum test (reported as Z) was used if the data did not meet the normality assumption. A mixed-effect model for repeated measures with genotype, and session as fixed effects and subject as a random effect was used to investigate percentage of correct responses during the VD task. Residuals of models were inspected for normality and an alpha level of p = 0.05 was used to determine significance in all statistical tests. For electrophysiological statistical testing, most data were non-normally distributed and nonparametric test were applied. For within-subject analysis the delta value between the two conditions was calculated and compared to zero. Significance was tested using the Wilcoxon signed-rank test (reported as Z). Correction for multiple comparison was performed using the Benjamini-Hochberg procedure with a false discovery rate threshold of *q* = 0.05^60,61^. For clear representation of the data all descriptive statistics, such as mean, median, SD, and ranges, together with p values and thresholds are presented in tables (either in the text or as supplementary material) and box plots with individual points for each subject are used, rather than commonly reported bars graphs^62^. Box plots represent the interquartile range; the solid line inside the box indicates the median, the box represents 50% of data points between the first and third quartile, and the upper and lower whiskers represent scores outside the middle 50%.

### *In Vitro* experimental methods

#### Animals

A separate, satellite group of *App*^*NL-G-F*^ male mice were group-housed in individually ventilated cages under a reversed 12–12 light cycle (lights off 07:00–19:00; light intensity ~100 lux) and controlled environmental conditions throughout the study (22 ± 2 °C ambient temperature and relative humidity at 60%). Home cages were equipped with corn cob bedding, a tinted polycarbonate shelter and tissue for nesting material. Food and water were provided *ad libitum*.

### Tissue collection and processing

Mice were euthanized by decapitation after which brains were excised. Olfactory lobes and hindbrain were removed from tissue processed for biochemistry. Brain hemispheres were weighed, immediately frozen on dry ice and stored at −80 °C prior to biochemical (right hemisphere) analysis. For the preparation of brain homogenates, tissue was thawed on ice in pre-cooled GuHCl extraction buffer (5 M Guanidin-hydrochloride, 50 mM Tris-HCl, pH 8.0, 1 mL/100 mg tissue). Homogenization was done utilizing 4.5 mL TallPrep tubes (MP Biomedicals) containing Lysing matrix D (1.4 mm ceramic beads) and FastPrep-24 5 G homogenizer (MP Biomedicals). Subsequently, homogenates were shaken for 3 hours at room temperature and stored at −80 °C until further use. The left hemisphere was post-fixed overnight in a formalin-based fixative, embedded in paraffin and sliced (5 µm) with a microtome.

### Brain Aβ1–42 elisa

Aβ1–42 in *App*^*NL-G-F*^ brain extracts were quantified in a one-step direct sandwich ELISA with capture antibody JRF/cAβ42/26 that specifically recognizes Aβ ending at amino acid 42, and detection with SULFOTAG™-labelled 3D6 antibody that recognizes Aβ starting with amino acid 1. Equal volumes of brain homogenates of different mice belonging to the same age group (4 to 6 mice per time point) were pooled and then further diluted 1:10 in casein buffer (0.1% casein in PBS). Next, samples were spun for 20 minutes at 20.000 g (4 °C) and supernatants was collected to be further diluted in 0.5 M GuHCl buffer to optimal dilutions for use in the assay. Human Aβ1–42 standard peptide (Anaspec, San Jose, CA, USA) was dissolved in DMSO at 0.1 mg/mL and stored at −80 °C. For use in ELISA, standards were diluted in 0.5 M GuHCl buffer from 20.000 pg/mL down to 0 pg/mL.

Capture antibody was diluted in PBS (1.5 µg/mL), coated onto Multi-array 96-well SECTOR plates (Meso Scale Discovery, L15XA, 30 µL/well) and incubated overnight at 4 °C. After 5 washes with PBS containing 0.5% Tween20 (wash buffer), the plates were blocked in 0.1% casein buffer (2 hours at room temperature while shaking) and washed again with wash buffer (5x). Prior to their addition to the plates, samples and standards were mixed in an equal volume of SULFOTAG™-labelled detection antibody (diluted in 0.1% casein buffer) and were subsequently incubated on the plates overnight at 4 °C. The next day, plates were washed 5 times with wash buffer, 2 × MSD Read Buffer T was added to the wells and plates were immediately read with the MESO SECTOR S 600 plate reader. Using Meso Scale software, raw signals were normalized against the standard curve.

### Immunohistochemistry

Following deparaffinization and rehydration of the sections, antigen retrieval was performed (10 minutes incubation in 70% formic acid) and endogenous peroxidase activity was blocked with 3% hydrogen peroxide. Samples were incubated overnight with biotinylated primary antibodies (JRF/cAβ42/26 2 µg/mL; JRF/AβN/25 1 µg/mL^63^), diluted in antibody diluent with background reducing components (DAKO, Glostrup, Denmark). After extensive washing, streptavidin-HRP solution (Vector Labs, Burlingame, CA, USA) was applied for 30 minutes, followed by chromogenic labelling with 3,3-diaminobenzidine (DAB, DAKO). Slides were counterstained with hematoxylin, dehydrated and permanently mounted (Vectamount, Vector Labs). Imaging was performed with a NanoZoomer slide scanner (Hamamatsu Photonics, Shizuoka, Japan) and analysed with Matlab/Phaedra. Regions-of-interest (ROIs) were manually delineated in accordance with the Franklin and Paxinos atlas^59^ and for each ROI the percentage of DAB-labelled area per total area was calculated.

### Statistical analysis

Statistical analyses were conducted using GraphPad Prism, version 8 (GraphPad Software, Inc.). Using the Shapiro-Wilk test, the distributions of dependent variables were assessed for meeting the assumption of normality required in parametric statistical tests. Multiple group comparisons were made using the Brown-Forsythe and Welch ANOVA with Dunnett’s T3 multiple comparisons test. Data were considered significant when p < 0.05 (one symbol p < 0.05, two symbols, p < 0.01, three symbols p < 0.001, four symbols p < 0.0001).

## Supplementary information

Supplementary Material

## References

[1] Scheltens P (2016). Alzheimer’s disease. Lancet.

[2] Querfurth HM, LaFerla FM (2011). Mechanisms of Disease Alzheimer’s. new engl J. Med..

[3] Nimmrich V, Draguhn A, Axmacher N (2015). Neuronal Network Oscillations in Neurodegenerative Diseases. NeuroMolecular Med..

[4] Başar E (2016). What does the broken brain say to the neuroscientist? Oscillations and connectivity in schizophrenia, Alzheimer’s disease, and bipolar disorder. Int. J. Psychophysiol..

[5] Friedman D, Honig LS, Scarmeas N (2013). Seizures and Epilepsy in Alzheimer’s Disease. CNS Neurosci Ther..

[6] Jack CR (2010). Hypothetical model of dynamic biomarkers of the Alzheimer’s pathological cascade. Lancet Neurol..

[7] Sasaguri H (2017). APP mouse models for Alzheimer’s disease preclinical studies. EMBO J..

[8] Jankowsky, J. L. & Zheng, H. Practical considerations for choosing a mouse model of Alzheimer’s disease. *Mol. Neurodegener*. 1–22 10.1186/s13024-017-0231-7 (2017). 10.1186/s13024-017-0231-7 PMC5741956 29273078

[9] Nilsson, P., Saito, T. & Saido, T. C. New Mouse Model of Alzheimer’ s. 16–19 (2014). 10.1021/cn500105p PMC4102956 24852598

[10] Born HA (2014). Genetic Suppression of Transgenic APP Rescues Hypersynchronous Network Activity in a Mouse Model of Alzeimer’s Disease. J. Neurosci..

[11] Palop JJ (2007). Aberrant Excitatory Neuronal Activity and Compensatory Remodeling of Inhibitory Hippocampal Circuits in Mouse Models of Alzheimer’s Disease. Neuron.

[12] Palop JJ, Mucke L (2010). Amyloid-Β-induced neuronal dysfunction in Alzheimer’s disease: From synapses toward neural networks. Nat. Neurosci..

[13] Busche MA, Konnerth A (2015). Neuronal hyperactivity - A key defect in Alzeimer’s disease?. Bioessays J..

[14] Goutagny R, Gu N, Cavanagh C, Jackson J, Chabot J (2013). Alterations in hippocampal network oscillations and theta – gamma coupling arise before A b overproduction in a mouse model of Alzheimer’ s disease..

[15] Poza, J. *et al*. Phase-Amplitude Coupling Analysis of Spontaneous EEG Activity in Alzheimer’s Disease. *IEEE* 2259–2262 10.1109/EMBC.2017.8037305 (2017). 10.1109/EMBC.2017.8037305 29060347

[16] Palop, J. J. & Mucke, L. Epilepsy and Cognitive Impairments in Alzheimer Disease. *Arch Neurol*. **66** (2009). 10.1001/archneurol.2009.15 PMC2812914 19204149

[17] Rice, H. C. *et al*. Secreted amyloid-b precursor protein functions as a GABA B R1a ligand to modulate synaptic transmission. *Science (80-.)*. **363** (2019). 10.1126/science.aao4827 PMC6366617 30630900

[18] Nicolas M, Hassan BA (2014). Amyloid precursor protein and neural development. Development.

[19] Saito T (2014). Single App knock-in mouse models of Alzheimer’s disease. Nat. Neurosci..

[20] Sakakibara Y, Sekiya M, Saito T, Saido TC, Iijima KM (2018). Cognitive and emotional alterations in App knock-in mouse models of Aβ amyloidosis. BMC Neurosci..

[21] Masuda A (2016). Cognitive deficits in single App knock-in mouse models. Neurobiol. Learn. Mem..

[22] Whyte LS (2018). Reduction in open field activity in the absence of memory deficits in the AppNL−G−F knock-in mouse model of Alzheimer’s disease. Behav. Brain Res..

[23] Latif-Hernandez A (2019). Subtle behavioral changes and increased prefrontal-hippocampal network synchronicity in APP NL−G−F mice before prominent plaque deposition. Behav. Brain Res..

[24] Brown R (2018). Circadian and Brain State Modulation of Network Hyperexcitability in Alzheimer’s Disease. eneuro.

[25] Horner AE (2013). The touchscreen operant platform for testing learning and memory in rats and mice. Nat. Protoc..

[26] Axmacher N (2010). Cross-frequency coupling supports multi-item working memory in the human hippocampus. Proc. Natl. Acad. Sci..

[27] Tort ABL (2008). Dynamic cross-frequency couplings of local field potential oscillations in rat striatum and hippocampus during performance of a T-maze task. Proc. Natl. Acad. Sci..

[28] Lisman JE, Jensen O (2013). The Theta-Gamma Neural Code. Neuron.

[29] Jobert M (2012). Guidelines for the Recording and Evaluation of Pharmaco-EEG Data in Man: The International Pharmaco-EEG Society (IPEG). Neuropsychobiology.

[30] Colgin LL (2009). Frequency of gamma oscillations routes flow of information in the hippocampus. Nature.

[31] Keren, G. *Between-or within-subjects design: A methodological dilemma. A Handbook for Data Analysis in the Behaviorial Sciences*. (2014).

[32] Mehla J (2019). Age-dependent behavioral and biochemical characterization of single APP knock-in mouse (APP NL-G-F / NL-G-F) model of Alzheimer’ s disease. Neurobiol. Aging.

[33] Palop JJ, Mucke L (2016). Network abnormalities and interneuron dysfunction in Alzheimer disease. Nat. Rev. Neurosci..

[34] LaFerla FM, Green KN (2012). Animal models of Alzheimer disease. Cold Spring Harb Perspect Med.

[35] Scheltens P (2016). Alzheimer’ s disease. Lancet.

[36] Webster SJ, Bachstetter AD, Nelson PT, Schmitt FA, Van Eldik LJ (2014). Using mice to model Alzheimer’s dementia: An overview of the clinical disease and the preclinical behavioral changes in 10 mouse models. Front. Genet..

[37] Serrano-Pozo A, Frosch MP, Masliah E, Hyman BT (2011). Neuropathological alterations in Alzheimer disease. Cold Spring Harb. Perspect. Med..

[38] Thorn CA, Atallah H, Howe M, Graybiel AM (2010). Differential dynamics of activity changes in dorsolateral and dorsomedial striatal loops during learning. Neuron.

[39] Canolty RT (2006). High Gamma Power Is Phase-Locked to Theta Oscillations in Human Neocortex. Science (80-.)..

[40] Lega B, Burke J, Jacobs J, Kahana MJ (2016). Slow-Theta-to-Gamma Phase-Amplitude Coupling in Human Hippocampus Supports the Formation of New Episodic Memories. Cereb. Cortex.

[41] Amemiya S, Redish AD (2018). Hippocampal Theta-Gamma Coupling Reflects State-Dependent Information Processing in Decision Making. Cell Rep..

[42] Belluscio MA, Mizuseki K, Schmidt R, Kempter R, Buzsáki G (2012). Cross-frequency phase-phase coupling between θ and γ oscillations in the hippocampus. J. Neurosci..

[43] Tort ABL, Komorowski RW, Manns JR, Kopell NJ, Eichenbaum H (2009). Theta-gamma coupling increases during the learning of item-context associations. Proc. Natl. Acad. Sci..

[44] Dimitriadis SI, Laskaris NA, Bitzidou MP, Tarnanas I, Tsolaki MN (2015). A novel biomarker of amnestic MCI based on dynamic cross-frequency coupling patterns during cognitive brain responses. Front. Neurosci..

[45] Tamura M, Spellman TJ, Rosen AM, Gogos JA, Gordon JA (2017). Hippocampal-prefrontal theta-gamma coupling during performance of a spatial working memory task. Nat. Commun..

[46] Iaccarino HF (2016). Gamma frequency entrainment attenuates amyloid load and modifies microglia. Nature.

[47] Klein AS, Donoso JR, Kempter R, Schmitz D, Beed P (2016). Early Cortical Changes in Gamma Oscillations in Alzheimer’s Disease. Front. Syst. Neurosci..

[48] Bazzigaluppi P (2018). Early‐stage attenuation of phase‐amplitude coupling in the hippocampus and medial prefrontal cortex in a transgenic rat model of Alzheimer’s disease. J. Neurochem..

[49] Engels MM (2015). Declining functional connectivity and changing hub locations in Alzheimer’s disease: an EEG study. BMC Neurol..

[50] Chen L, Saito T, Saido TC, Mody I (2018). Novel Quantitative Analyses of Spontaneous Synaptic Events in Cortical Pyramidal Cells Reveal Subtle Parvalbumin-Expressing Interneuron Dysfunction in a Knock-In Mouse Model of Alzheimer’s Disease. eneuro.

[51] Nakazono, T., Jun, H., Blurton-Jones, M., Green, K. N. & Igarashi, K. M. Gamma oscillations in the entorhinal-hippocampal circuit underlying memory and dementia. *Neurosci. Res*. 10.1016/j.neures.2018.02.002 (2018). 10.1016/j.neures.2018.02.002 PMC7059556 29438778

[52] Shah D (2018). Spatial reversal learning defect coincides with hypersynchronous telencephalic BOLD functional connectivity in APPNL-F/NL-F knock-in mice. Sci. Rep..

[53] Voevodskaya O (2018). Altered structural network organization in cognitively normal individuals with amyloid pathology. Neurobiol. Aging.

[54] Coben LA, Danziger W, Storandt M (1985). A longitudinal EEG study of mild senile dementia of Alzheimer type: changes at 1 year and at 2.5 years. Electroencephalogr. Clin. Neurophysiol..

[55] Fischer, C. E. *et al*. Determining the impact of psychosis on rates of false-positive and false-negative diagnosis in Alzheimer’s disease. In *Alzheimer’s and Dementia: Translational Research and Clinical Interventions* **3**, 385–392 (Elsevier Inc., 2017). 10.1016/j.trci.2017.06.001 PMC5651446 29067344

[56] Vossel KA (2016). Incidence and impact of subclinical epileptiform activity in Alzheimer’s disease. Ann. Neurol..

[57] Vossel KA (2013). Seizures and epileptiform activity in the early stages of Alzheimer disease. JAMA Neurol..

[58] Lam AD (2017). Silent hippocampal seizures and spikes identified by foramen ovale electrodes in Alzheimer’s disease. Nat. Med..

[59] Franklin, K. B., & Paxinos, G. *The mouse brain in stereotaxic coordinates*. (Academic press., 1997).

[60] Benjamini & Hochberg, Y. Controlling the False Discovery Rate: A Practical and Powerful Approach to Multiple Testing. *J. R. Stat. Soc. Ser. B* 289–300 (1995).

[61] Glickman ME, Rao SR, Schultz MR (2014). False discovery rate control is a recommended alternative to Bonferroni-type adjustments in health studies. Journal of Clinical Epidemiology.

[62] Weissgerber TL, Milic NM, Winham SJ, Garovic VD (2015). Beyond bar and line graphs: time for a new data presentation paradigm. PLoS Biol..

[63] Mercken M (2000). Specific ELISA systems for the detection of endogenous human and rodent ABETA40 and ABETA42. Neurobiol. Aging.

