# Supplementary material for: Neural oscillations during cognitive processes in an App knock-in mouse model of Alzheimer’s disease pathology
Source: Sci Rep. 2019 Nov 8;9:16363. doi: 10.1038/s41598-019-51928-w (PMC6841667; doi:10.1038/s41598-019-51928-w)
Supplement: Supplementary file 1 — Supplementary Material [file 41598_2019_51928_MOESM1_ESM.pdf]

**SUPPLEMENTARY MATERIAL**

# Neural oscillations during cognitive processes in an *App* knock-in mouse model of Alzheimer's disease pathology.

**Sofia Jacob[1,2], Gethin Davies[1], Marijke De Bock[1], Bart Hermans[1], Cindy Wintmolders[1], Astrid Bottelbergs[1], Marianne Borgers[1], Clara Theunis[1], Bianca Van Broeck[1], Nikolay V. Manyakov[3], Detlef Balschun[2], Wilhelmus H.I.M. Drinkenburg[1,4*]**

[1] Department of Neuroscience, Janssen Research & Development, a Division of Janssen Pharmaceutica NV Beerse, Belgium

[2]Brain & Cognition, KU Leuven, Leuven, Belgium

[3]Digital Phenotyping, Janssen Research & Development, a Division of Janssen Pharmaceutica NV, Beerse, Belgium

[4] Groningen Institute for Evolutionary Life Sciences, University of Groningen, The Netherlands

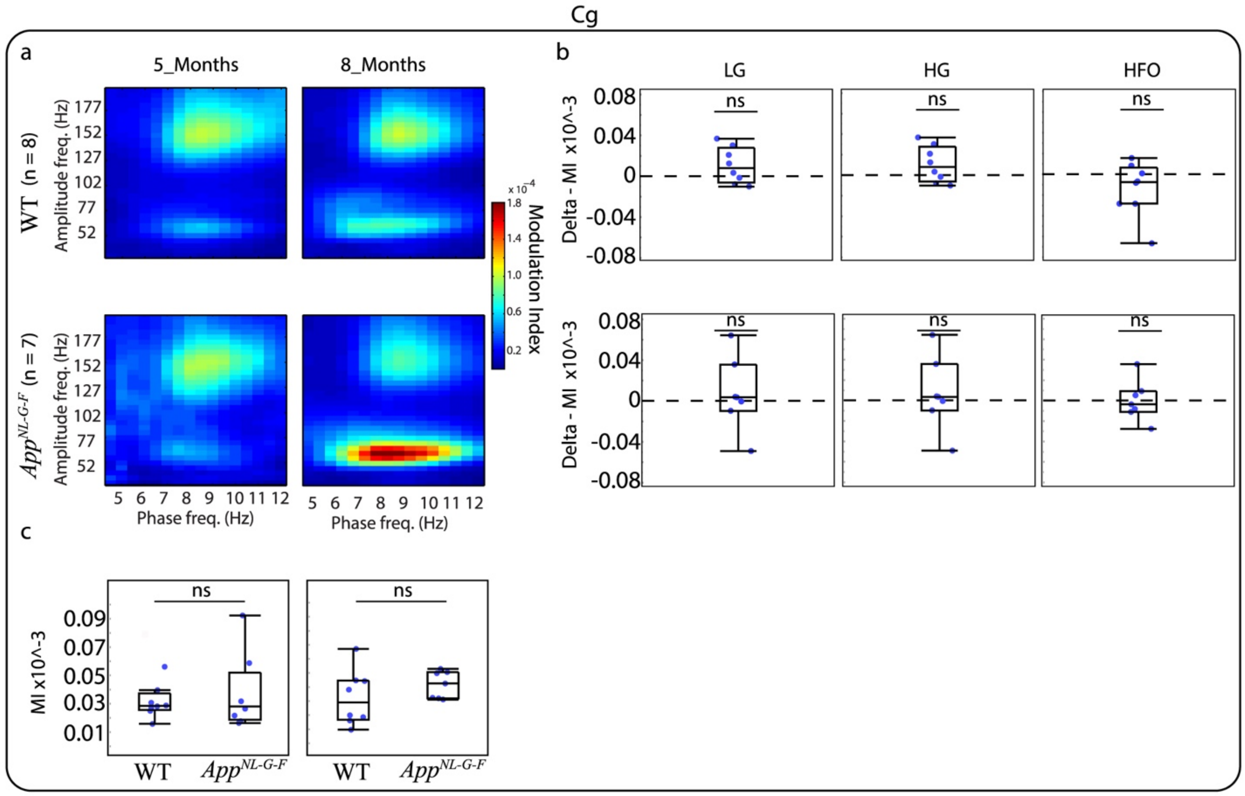


**Supplementary figure 1**. Mean phase-amplitude coupling (PAC) during home-cage environment exploration for the cingulate cortex (Cg). a. Phase-amplitude comodulograms plotted for WT mice (top panels) and *App*$^{NL\text{-}G\text{-}F}$mice (lower panels) for 5 months (left) and 8 months of age (right). b. Modulation index (MI) delta between 8 and 5 months for low gamma (LG, left), high gamma (HG, middle) and high frequency oscillations (HFO, right) for WT mice (top panels) and *App*$^{NL\text{-}G\text{-}F}$mice (lower panels). Comparison at each frequency band showed no significant difference for both WT and *App*$^{NL\text{-}G\text{-}F}$ mice. c. MI between WT and *App*$^{NL\text{-}G\text{-}F}$mice for 5 months (left) and 8 months (right) of age. Comparison at each frequency band showed no significant difference. Values are represented in box plots were individual points indicate (b) delta or (c) absolute values for each subject. Significance level at $q = 0.05$, false discovery rate (FSD) corrected for multiple comparisons. ns: non-significant.

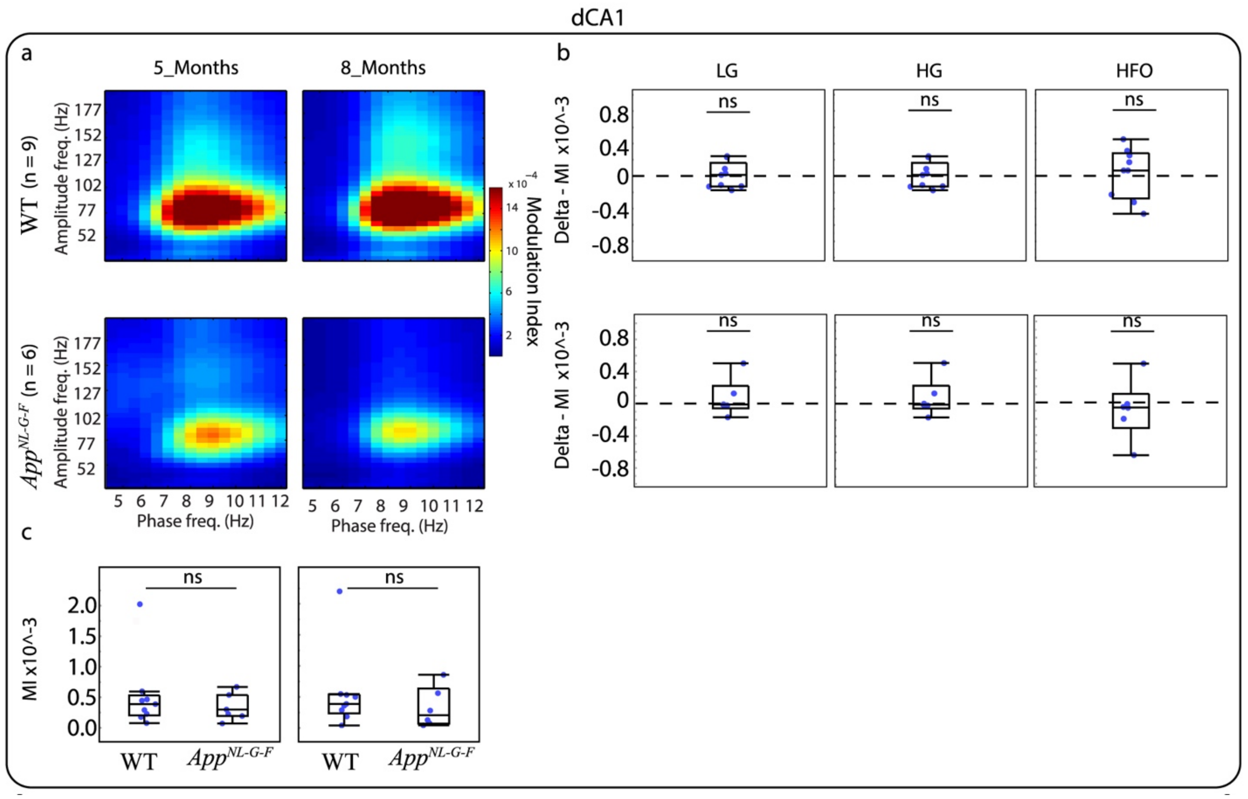


**Supplementary figure 2**. Mean phase-amplitude coupling (PAC) during home-cage environment exploration for the dorsal CA1 region of the hippocampus (dCA1). a. Phase-amplitude comodulograms plotted for WT mice (top panels) and *App*$^{NL\text{-}G\text{-}F}$ mice (lower panels) for 5 months (left) and 8 months of age (right). b. Modulation index (MI) delta between 8 and 5 months for low gamma (LG, left), high gamma (HG, middle) and high frequency oscillations (HFO, right) for WT mice (top panels) and *App*$^{NL\text{-}G\text{-}F}$mice (lower panels). Comparison at each frequency band showed no significant difference for both WT and *App*$^{NL\text{-}G\text{-}F}$mice. c. MI between WT and *App*$^{NL\text{-}G\text{-}F}$mice for 5 months (left) and 8 months (right) of age. Comparison at each frequency band showed no significant difference. Values are represented in box plots were individual points indicate (b) delta or (c) absolute values for each subject. Significance level at $q$ = 0.05, false discovery rate (FSD) corrected for multiple comparisons. ns: non-significant.

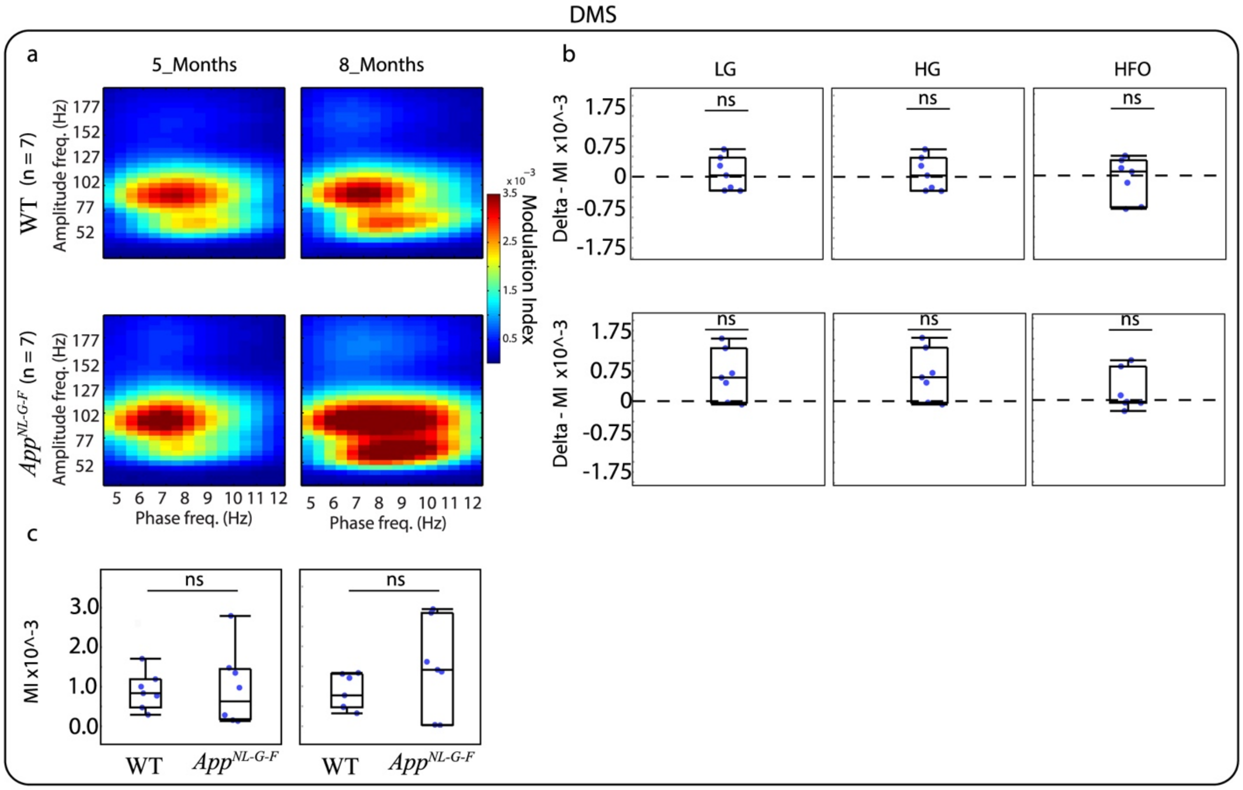


**Supplementary figure 3**. Mean phase-amplitude coupling (PAC) during home-cage environment exploration for the dorsal medial striatum (DMS). a. Phase-amplitude comodulograms plotted for WT mice (top panels) and *App*$^{NL\text{-}G\text{-}F}$ mice (lower panels) for 5 months (left) and 8 months of age (right). b. Modulation index (MI) delta between 8 and 5 months for low gamma (LG, left), high gamma (HG, middle) and high frequency oscillations (HFO, right) for WT mice (top panels) and *App*$^{NL\text{-}G\text{-}F}$mice (lower panels). Comparison at each frequency band showed no significant difference for both WT and *App*$^{NL\text{-}G\text{-}F}$mice. c. MI between WT and *App*$^{NL\text{-}G\text{-}F}$mice for 5 months (left) and 8 months (right) of age. Comparison at each frequency band showed no significant difference. Values are represented in box plots were individual points indicate (b) delta or (c) absolute values for each subject. Significance level at $q = 0.05$, false discovery rate (FSD) corrected for multiple comparisons. ns: non-significant.

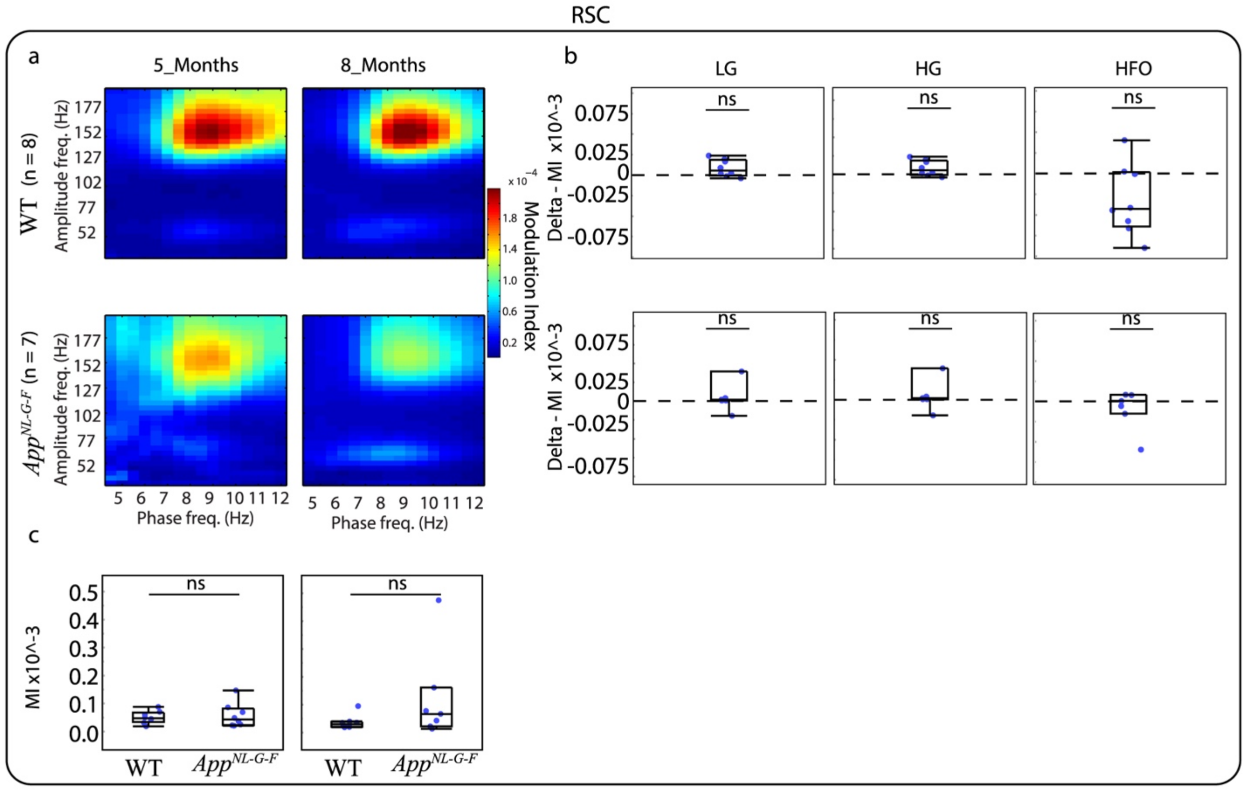


**Supplementary figure 4**. Mean phase-amplitude coupling (PAC) during home cage environment exploration for the retrosplenial cortex (RSC). a. Phase-amplitude comodulograms plotted for WT mice (top panels) and *App*$^{NL\text{-}G\text{-}F}$mice (lower panels) for 5 months (left) and 8 months of age (right). b. Modulation index (MI) delta between 8 and 5 months for low gamma (LG, left), high gamma (HG, middle) and high frequency oscillations (HFO, right) for WT mice (top panels) and *App*$^{NL\text{-}G\text{-}F}$mice (lower panels). Comparison at each frequency band showed no significant difference for both WT and *App*$^{NL\text{-}G\text{-}F}$mice. c. MI between WT and *App*$^{NL\text{-}G\text{-}F}$mice for 5 months (left) and 8 months (right) of age. Comparison at each frequency band showed no significant difference. Values are represented in box plots were individual points indicate (b) delta or (c) absolute values for each subject. Significance level at $q = 0.05$, false discovery rate (FSD) corrected for multiple comparisons. ns: non-significant.

Supplementary table 1. Between-subject analysis of relative power spectral density (PSD) during the visual discrimination (VD) task.

| Condition | Brain Area | Freq | Z | Prob > [Z] | p value rank | FDR threshold |
|---|---|---|---|---|---|---|
| Task_Start | Cg | Theta | 2.02523 | 0.04280 | 1 | 0.00208 |
| | | Gamma | -1.44659 | 0.14800 | 7 | 0.01458 |
| | | HFO | -1.67805 | 0.09300 | 5 | 0.01042 |
| | dCA1 | Theta | 1.78968 | 0.07350 | 3 | 0.00625 |
| | | Gamma | -1.68121 | 0.08270 | 4 | 0.00833 |
| | | HFO | 1.3558 | 0.17520 | 9 | 0.01875 |
| | DMS | Theta | 0.6365 | 0.52440 | 17 | 0.03542 |
| | | Gamma | 0 | 1.00000 | 24 | 0.05000 |
| | | HFO | -0.40505 | 0.68540 | 20 | 0.04167 |
| | RSC | Theta | 0.84681 | 0.39710 | 14 | 0.02917 |
| | | Gamma | -0.1058 | 0.91570 | 22 | 0.04583 |
| | | HFO | -0.84681 | 0.39710 | 13 | 0.02708 |
| Task_End | Cg | Theta | 1.56232 | 0.11820 | 6 | 0.01250 |
| | | Gamma | -1.21514 | 0.22430 | 11 | 0.02292 |
| | | HFO | -1.79378 | 0.07280 | 2 | 0.00417 |
| | dCA1 | Theta | 0.70502 | 0.48080 | 16 | 0.03333 |
| | | Gamma | -1.03042 | 0.30280 | 12 | 0.02500 |
| | | HFO | 1.24735 | 0.21230 | 10 | 0.02083 |
| | DMS | Theta | -0.75223 | 0.45190 | 15 | 0.03125 |
| | | Gamma | 0.28932 | 0.77230 | 21 | 0.04375 |
| | | HFO | 0.05786 | 0.95390 | 23 | 0.04792 |
| | RSC | Theta | 0.63511 | 0.52540 | 19 | 0.03958 |
| | | Gamma | -0.63511 | 0.52540 | 18 | 0.03750 |
| | | HFO | -1.37607 | 0.16880 | 8 | 0.01667 |

Conditions: Task_Start = start of the VD task, Task_End = end of the VD task.
Brain areas: Cingulate Cortex (Cg), dorsal CA1 region of the hippocampus (dCA1), dorsomedial striatum (DMS), and retrosplenial cortex (RSC).
Frequencies: theta, gamma, and high frequency oscillations (HFO).
Z: Wilcoxon rank-sum test.
Significance level at $q = 0.05$, false discovery rate (FSD) corrected for multiple comparisons.

Supplementary table 2. Relative power spectral density (PSD, μV^2/Hz) during the no-task condition.

| Genotype | Brain Area | Freq | Condition | Mean | SD | Median | Range | N | Z | Prob > [Z] | p value rank | FDR threshold |
|---|---|---|---|---|---|---|---|---|---|---|---|---|
| WT | Cg | Theta | 5_Months | 0.032498 | 0.005879 | 0.032734 | 0.016401 | 8 | 7.00 | 0.3828 | 14 | 0.0292 |
| | | | 8_Months | 0.035089 | 0.003226 | 0.034196 | 0.009341 | | | | | |
| | | Gamma | 5_Months | 0.004650 | 0.000881 | 0.004627 | 0.002497 | | -13.00 | 0.0781 | 2 | 0.0042 |
| | | | 8_Months | 0.003925 | 0.000508 | 0.003733 | 0.001252 | | | | | |
| | | HFO | 5_Months | 0.000903 | 0.000216 | 0.000863 | 0.000502 | | -10.00 | 0.1953 | 7 | 0.0146 |
| | | | 8_Months | 0.000751 | 0.000137 | 0.000755 | 0.000410 | | | | | |
| $App^{NL-G-F}$ | | Theta | 5_Months | 0.035439 | 0.004817 | 0.035170 | 0.014066 | 7 | -4.00 | 0.5781 | 17 | 0.0354 |
| | | | 8_Months | 0.032385 | 0.003577 | 0.033203 | 0.008848 | | | | | |
| | | Gamma | 5_Months | 0.004493 | 0.000512 | 0.004522 | 0.001353 | | -9.00 | 0.1563 | 5 | 0.0104 |
| | | | 8_Months | 0.004375 | 0.000888 | 0.004264 | 0.002362 | | | | | |
| | | HFO | 5_Months | 0.000819 | 0.000153 | 0.000793 | 0.000416 | | 8.00 | 0.2188 | 8 | 0.0167 |
| | | | 8_Months | 0.000872 | 0.000122 | 0.000885 | 0.000366 | | | | | |
| WT | dCA1 | Theta | 5_Months | 0.048081 | 0.008695 | 0.049213 | 0.030641 | 9 | -8.50 | 0.3594 | 12 | 0.0250 |
| | | | 8_Months | 0.046197 | 0.006613 | 0.044063 | 0.019754 | | | | | |
| | | Gamma | 5_Months | 0.002954 | 0.000694 | 0.002938 | 0.002094 | | 5.50 | 0.5703 | 16 | 0.0333 |
| | | | 8_Months | 0.003319 | 0.000642 | 0.003210 | 0.001863 | | | | | |
| | | HFO | 5_Months | 0.000232 | 0.000063 | 0.000224 | 0.000204 | | 3.50 | 0.7344 | 18 | 0.0375 |
| | | | 8_Months | 0.000240 | 0.000052 | 0.000252 | 0.000143 | | | | | |
| $App^{NL-G-F}$ | | Theta | 5_Months | 0.051162 | 0.008747 | 0.052458 | 0.026774 | 6 | -8.50 | 0.0938 | 3 | 0.0063 |
| | | | 8_Months | 0.046925 | 0.009736 | 0.042482 | 0.024645 | | | | | |
| | | Gamma | 5_Months | 0.003012 | 0.000665 | 0.002945 | 0.001758 | | 7.50 | 0.1563 | 6 | 0.0125 |
| | | | 8_Months | 0.003344 | 0.000697 | 0.003593 | 0.001737 | | | | | |
| | | HFO | 5_Months | 0.000262 | 0.000077 | 0.000251 | 0.000206 | | 4.50 | 0.4375 | 15 | 0.0313 |
| | | | 8_Months | 0.000276 | 0.000081 | 0.000301 | 0.000215 | | | | | |
| WT | DMS | Theta | 5_Months | 0.045772 | 0.004106 | 0.044150 | 0.010752 | 7 | -7.00 | 0.2969 | 11 | 0.0229 |
| | | | 8_Months | 0.042794 | 0.005472 | 0.044168 | 0.016964 | | | | | |
| | | Gamma | 5_Months | 0.003815 | 0.000781 | 0.003851 | 0.001884 | | 0.00 | 1.0000 | 24 | 0.0500 |
| | | | 8_Months | 0.003626 | 0.000823 | 0.003884 | 0.002297 | | | | | |
| | | HFO | 5_Months | 0.000314 | 0.000037 | 0.000308 | 0.000107 | | -8.00 | 0.2188 | 9 | 0.0188 |
| | | | 8_Months | 0.000266 | 0.000092 | 0.000251 | 0.000298 | | | | | |
| $App^{NL-G-F}$ | | Theta | 5_Months | 0.044840 | 0.007417 | 0.041795 | 0.019151 | 7 | -9.00 | 0.1563 | 4 | 0.0083 |
| | | | 8_Months | 0.037259 | 0.007511 | 0.036160 | 0.021080 | | | | | |
| | | Gamma | 5_Months | 0.004054 | 0.000800 | 0.003906 | 0.002012 | | 12.00 | 0.0469 | 1 | 0.0021 |
| | | | 8_Months | 0.004780 | 0.001040 | 0.004386 | 0.002508 | | | | | |
| | | HFO | 5_Months | 0.000351 | 0.000104 | 0.000318 | 0.000319 | | 6.00 | 0.3750 | 13 | 0.0271 |
| | | | 8_Months | 0.000433 | 0.000260 | 0.000320 | 0.000559 | | | | | |
| WT | RSC | Theta | 5_Months | 0.032721 | 0.005933 | 0.032494 | 0.015763 | 8 | -9.00 | 0.2500 | 10 | 0.0208 |
| | | | 8_Months | 0.028861 | 0.002459 | 0.029072 | 0.007262 | | | | | |

| | | | | | | | | | | | | |
|---|---|---|---|---|---|---|---|---|---|---|---|---|
| | | Gamma | 5_Months | 0.004397 | 0.000705 | 0.004731 | 0.001831 | | -1.00 | 0.9453 | 21 | 0.0438 |
| | | | 8_Months | 0.004471 | 0.000730 | 0.004296 | 0.002552 | | | | | |
| | | HFO | 5_Months | 0.000941 | 0.000209 | 0.000957 | 0.000512 | | 1.00 | 0.9453 | 22 | 0.0458 |
| | | | 8_Months | 0.000950 | 0.000224 | 0.000978 | 0.000578 | | | | | |
| *App$^{NL-G-F}$* | | Theta | 5_Months | 0.034067 | 0.006545 | 0.032434 | 0.019512 | 7 | 0.00 | 1.0000 | 23 | 0.0479 |
| | | | 8_Months | 0.034066 | 0.008875 | 0.035409 | 0.024101 | | | | | |
| | | Gamma | 5_Months | 0.004359 | 0.000695 | 0.004425 | 0.001937 | | -2.00 | 0.8125 | 19 | 0.0396 |
| | | | 8_Months | 0.004259 | 0.000954 | 0.004073 | 0.002347 | | | | | |
| | | HFO | 5_Months | 0.000882 | 0.000160 | 0.000886 | 0.000494 | | -1.00 | 0.9375 | 20 | 0.0417 |
| | | | 8_Months | 0.000757 | 0.000374 | 0.000916 | 0.000805 | | | | | |

Brain areas: Cingulate Cortex (Cg), dorsal CA1 region of the hippocampus (dCA1), dorsomedial striatum (DMS), and retrosplenial cortex (RSC).
Frequencies: theta, gamma, and high frequency oscillations (HFO).
Conditions: 5 and 8 months of age.
Z: Wilcoxon rank-sum test.
Significance level at $q = 0.05$, false discovery rate (FSD) corrected for multiple comparisons.

Supplementary table 3. Phase-amplitude coupling (PAC) during the no-task condition.

| Genotype | Brain Area | Freq | Condition | Mean | SD | Median | Range | N | Z | Prob > [Z] | p value rank | FDR threshold |
|---|---|---|---|---|---|---|---|---|---|---|---|---|
| WT | Cg | LG | 5_Months | 0.00001604 | 0.00000679 | 0.00001735 | 0.00001969 | 8 | 10 | 0.1953 | 8 | 0.0167 |
| | | | 8_Months | 0.00002658 | 0.00001878 | 0.00001970 | 0.00005335 | | | | | |
| | | HG | 5_Months | 0.00002065 | 0.00001910 | 0.00001346 | 0.00005486 | | -8 | 0.3125 | 11 | 0.0229 |
| | | | 8_Months | 0.00005186 | 0.00006217 | 0.00002513 | 0.00016830 | | | | | |
| | | HFO | 5_Months | 0.00004043 | 0.00002254 | 0.00003905 | 0.00007542 | | 10 | 0.1953 | 9 | 0.0188 |
| | | | 8_Months | 0.00002622 | 0.00001665 | 0.00002188 | 0.00004955 | | | | | |
| *App*$^{NL-G-F}$ | | LG | 5_Months | 0.00001883 | 0.00019200 | 0.00001100 | 0.00005415 | 8 | 3 | 0.6875 | 12 | 0.0250 |
| | | | 8_Months | 0.00002609 | 0.00002387 | 0.00001302 | 0.00006454 | | | | | |
| | | HG | 5_Months | 0.00002902 | 0.00001965 | 0.00002507 | 0.00006313 | | -1 | 0.9375 | 18 | 0.0375 |
| | | | 8_Months | 0.00004248 | 0.00004373 | 0.00002422 | 0.00011190 | | | | | |
| | | HFO | 5_Months | 0.00004543 | 0.00003353 | 0.00003605 | 0.00009644 | | 3 | 0.6875 | 13 | 0.0271 |
| | | | 8_Months | 0.00004594 | 0.00002860 | 0.00004744 | 0.00007710 | | | | | |
| WT | dCA1 | LG | 5_Months | 0.00029700 | 0.00028960 | 0.00021690 | 0.00095190 | 9 | 0.5 | 1.0000 | 23 | 0.0479 |
| | | | 8_Months | 0.00030550 | 0.00024820 | 0.00023210 | 0.00085270 | | | | | |
| | | HG | 5_Months | 0.00106080 | 0.00140600 | 0.00060480 | 0.00462340 | | 2.5 | 0.8203 | 17 | 0.0354 |
| | | | 8_Months | 0.00113750 | 0.00128750 | 0.00082610 | 0.00436190 | | | | | |
| | | HFO | 5_Months | 0.00037210 | 0.00036460 | 0.00022020 | 0.00118140 | | 0.5 | 1.0000 | 24 | 0.0500 |
| | | | 8_Months | 0.00040580 | 0.00050110 | 0.00023170 | 0.00167640 | | | | | |
| *App*$^{NL-G-F}$ | | LG | 5_Months | 0.00014490 | 0.00008364 | 0.00018970 | 0.00019500 | 7 | -0.5 | 1.0000 | 21 | 0.0438 |
| | | | 8_Months | 0.00020730 | 0.00026040 | 0.00012890 | 0.00070540 | | | | | |
| | | HG | 5_Months | 0.00059030 | 0.00044000 | 0.00041590 | 0.00108500 | | -5.5 | 0.3125 | 10 | 0.0208 |
| | | | 8_Months | 0.00060730 | 0.00061780 | 0.00045360 | 0.00158750 | | | | | |
| | | HFO | 5_Months | 0.00031120 | 0.00027890 | 0.00019890 | 0.00072340 | | -0.05 | 1.0000 | 22 | 0.0458 |
| | | | 8_Months | 0.00024660 | 0.00024630 | 0.00013980 | 0.00053900 | | | | | |
| WT | DMS | LG | 5_Months | 0.00045270 | 0.00022320 | 0.00049090 | 0.00057080 | 7 | 3 | 0.6875 | 15 | 0.0313 |
| | | | 8_Months | 0.00052680 | 0.00024900 | 0.00039710 | 0.00056540 | | | | | |
| | | HG | 5_Months | 0.00184980 | 0.00110680 | 0.00199810 | 0.00330100 | | -1 | 0.9375 | 20 | 0.0417 |
| | | | 8_Months | 0.00179890 | 0.00084500 | 0.00168740 | 0.00210650 | | | | | |
| | | HFO | 5_Months | 0.00064920 | 0.00032630 | 0.00067930 | 0.00091810 | | 3 | 0.6875 | 16 | 0.0333 |
| | | | 8_Months | 0.00056410 | 0.00036110 | 0.00054390 | 0.00096120 | | | | | |
| *App*$^{NL-G-F}$ | | LG | 5_Months | 0.00033170 | 0.00035940 | 0.00020280 | 0.00107820 | 8 | 11 | 0.0781 | 1 | 0.0021 |
| | | | 8_Months | 0.00099570 | 0.00095130 | 0.00081650 | 0.00242700 | | | | | |
| | | HG | 5_Months | 0.00182690 | 0.00175710 | 0.00127930 | 0.00502120 | | 3 | 0.6875 | 14 | 0.0292 |
| | | | 8_Months | 0.00287100 | 0.00225590 | 0.00285780 | 0.00558370 | | | | | |
| | | HFO | 5_Months | 0.00075120 | 0.00077650 | 0.00052170 | 0.00221140 | | 11 | 0.0781 | 2 | 0.0042 |
| | | | 8_Months | 0.00104090 | 0.00080920 | 0.00105910 | 0.00199000 | | | | | |
| WT | RSC | LG | 5_Months | 0.00000963 | 0.00000824 | 0.00000731 | 0.00002398 | 8 | 13 | 0.0781 | 3 | 0.0063 |
| | | | 8_Months | 0.00001805 | 0.00001521 | 0.00001445 | 0.00004503 | | | | | |

| | | | | | | | | | | | | |
|---|---|---|---|---|---|---|---|---|---|---|---|---|
| | | HG | 5_Months | 0.00000716 | 0.00000390 | 0.00000564 | 0.00000967 | | -13 | 0.0781 | 5 | 0.0104 |
| | | | 8_Months | 0.00002254 | 0.00002640 | 0.00001188 | 0.00007954 | | | | | |
| | | HFO | 5_Months | 0.00008053 | 0.00003823 | 0.00007478 | 0.00011690 | | 13 | 0.0781 | 4 | 0.0083 |
| | | | 8_Months | 0.00004843 | 0.00004582 | 0.00003461 | 0.00014290 | | | | | |
| *App*$^{NL-G-F}$ | | LG | 5_Months | 0.00001497 | 0.00001558 | 0.00001039 | 0.00004568 | 8 | 9 | 0.1563 | 6 | 0.0125 |
| | | | 8_Months | 0.00004742 | 0.00007581 | 0.00000769 | 0.00020140 | | | | | |
| | | HG | 5_Months | 0.00002339 | 0.00002322 | 0.00001764 | 0.00006692 | | 1 | 0.9375 | 19 | 0.0396 |
| | | | 8_Months | 0.00022050 | 0.00046280 | 0.00000988 | 0.00124630 | | | | | |
| | | HFO | 5_Months | 0.00008308 | 0.00006097 | 0.00006674 | 0.00018000 | | 9 | 0.1563 | 7 | 0.0146 |
| | | | 8_Months | 0.00010670 | 0.00007750 | 0.00010070 | 0.00022860 | | | | | |

Brain areas: Cingulate Cortex (Cg), dorsal CA1 region of the hippocampus (dCA1), dorsomedial striatum (DMS), and retrosplenial cortex (RSC).
Frequencies: theta, gamma and high frequency oscillations (HFO).
Conditions: 5 and 8 months of age.
Descriptive statistics for Modulation Index (MI), N: sample size, Z: Wilcoxon rank-sum test.
Significance level at $q = 0.05$, false discovery rate (FSD) corrected for multiple comparisons.
Grey cells indicated significance values. P value ranks lower than 8 are statistically significant different between the two conditions.
